# SARS-CoV-2 Mutations and Their Impact on Diagnostics, Therapeutics and Vaccines

**DOI:** 10.3389/fmed.2022.815389

**Published:** 2022-02-22

**Authors:** Suresh Thakur, Shalitha Sasi, Sindhu Gopinathan Pillai, Ayantika Nag, Dhananjay Shukla, Ritu Singhal, Sameer Phalke, G. S. K. Velu

**Affiliations:** ^1^Trivitron Healthcare Pvt., Ltd., Visakhapatnam, India; ^2^Blue Horizon International Therapeutic Sciences, Hackensack, NJ, United States; ^3^Symbiosis Institute of Health Science, Symbiosis International University, Pune, India; ^4^MP Advisors, Vadodara, India; ^5^Department of Biotechnology, Guru Ghasidas Vishwavidyalaya (A Central University), Bilaspur, India; ^6^Department of Microbiology, National Institute of Tuberculosis and Respiratory Disease, New Delhi, India

**Keywords:** SARS-CoV-2, variants of concern, omicron, mutations, spike protein, diagnostics, vaccine

## Abstract

With the high rate of COVID-19 infections worldwide, the emergence of SARS-CoV-2 variants was inevitable. Several mutations have been identified in the SARS-CoV-2 genome, with the spike protein as one of the mutational hot spots. Specific amino acid substitutions such as D614G and N501Y were found to alter the transmissibility and virulence of the virus. The WHO has classified the variants identified with fitness-enhancing mutations as variants of concern (VOC), variants of interest (VOI) or variants under monitoring (VUM). The VOCs pose an imminent threat as they exhibit higher transmissibility, disease severity and ability to evade vaccine-induced and natural immunity. Here we review the mutational landscape on the SARS-CoV-2 structural and non-structural proteins and their impact on diagnostics, therapeutics and vaccines. We also look at the effectiveness of approved vaccines, antibody therapy and convalescent plasma on the currently prevalent VOCs, which are B.1.17, B.1.351, P.1, B.1.617.2 and B.1.1.529. We further discuss the possible factors influencing mutation rates and future directions.

## Introduction

Viruses evolve rapidly, and newly emerging viruses have been a major cause of public health concern several times in the history of humankind. Over the last two decades, we have witnessed epidemic situations caused by several viruses, including the severe acute respiratory syndrome coronavirus (SARS-CoV) in 2002, H1N1 influenza in 2009, the Middle East respiratory syndrome coronavirus (MERS-CoV) in 2012, Ebola virus disease (EVD) in 2013, and Zika virus in 2015, Nipah virus in 2018 and most recently the SARS-CoV2 virus (1, 2).

The first reported case of COVID-19 (Coronavirus disease–2019) was in December 2019. Consequently, the SARS-CoV-2 virus rapidly spread across the globe, resulting in an unprecedented pandemic situation, as announced by the World Health Organization (WHO) in March 2020. Since then, several countries have been hit by multiple waves of the COVID-19 pandemic that collapsed health care systems and halted economic activities. To date, a cohesive global effort is underway to bring down the transmission rates, save the vulnerable population and prevent further socio-economic damages. The world witnessed a rapid development of diagnostics, drugs and vaccines to track and tackle the pandemic. Although the arrival of vaccines has been the most potent weapon to combat the pandemic, several challenges remain. One of the most imminent threats is the emergence of viral variants. The disparity in region-wise vaccination rates is deemed a potential risk for the foreseeable future. For instance, higher incidences in low economic zones imply a higher mutation rate and a higher risk of new virulent mutants, which can again spread globally. The emergence of the more virulent variants of concern (VOCs) echoes this reality. Two years into the pandemic, many infections and deaths are still reported. As of December 2021, the delta variant is the most prevalent, with the highest infectivity rate compared to previous variants. Meanwhile, the infection numbers for the omicron variant identified in November 2021 are steadily on the rise. As of writing this paper, omicron is speculated to be more transmissible than delta. However, this has alerted the world that the pandemic is far from over, and new variants can still emerge. Therefore, further mutations in the genome that can translate into viral adaptability or increased pathogenicity can severely impact the current vaccination strategies, diagnostics, therapeutics and herd immunity.

**Figure 1:**
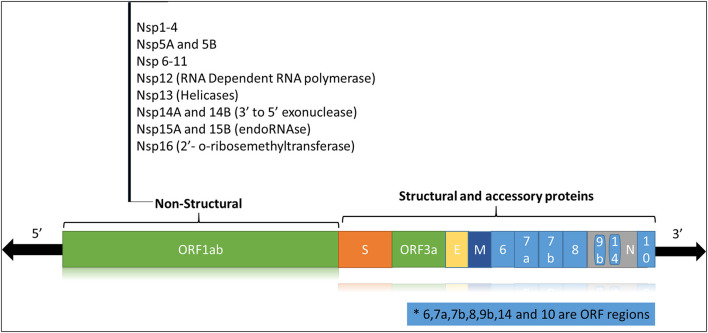
Schematic representation of the SARS-CoV-2 viral genome.

The SARS-CoV2 is a zoonotic RNA virus belonging to the family Coronaviridae, subfamily Coronavirinae, genus Beta-coronaviruses, and 2B lineage (3). Bats are the main natural reservoir for the viruses of this genus, and SARS-CoV-2 is thought to be naturally evolved from Bat CoVs (4). Like other coronaviruses, the SARS-CoV-2 genome consists of a positive-sense single-stranded RNA (+ssRNA) of ~29 Kb with a 5′-cap and 3'-UTR poly(A) tail. The viral genome is stabilized by the nucleocapsid protein (N) and enveloped in a bilipid structure comprising the membrane protein (M), spike protein (S) and envelope protein (E). The +ssRNA strand (Figure 1) has 14 open reading frames (ORFs) coding for

- Structural proteins (N, S, E, M),
- Non-structural proteins or nsps (ORF1 and ORF1ab) required for viral replication and assembly
- Accessory proteins (ORF3, ORF4a, ORF4b, ORF5, ORF8).

Genetic sequencing studies have revealed numerous mutations, mainly single nucleotide polymorphisms (SNPs) and insertion/deletions (indels), that are mostly neutral or mildly deleterious. However, a small percentage of mutations can alter the fitness and help the virus to adapt. These substitutions or deletions can alter the peptide polarity, affecting the structure and functionality of viral proteins involved in pathogenicity, infectivity, transmissibility and antigenicity. In order to classify the rapidly growing genome sequences and to track the real-time epidemiology and genetic evolution of SARS-CoV-2, several databases and nomenclature systems have come into place. The Global Initiative on Sharing All Influenza Data (GISAID; https://www.epicov.org) provides a comprehensive view of millions of globally available SARS-CoV-2 whole genome sequences and the significant mutations and variants identified to date (https://covariants.org/). GISAID nomenclature categorizes genomes into clades (based on marker mutations) that help understand large-scale diversity patterns and geographical spread (https://www.gisaid.org/references/statements-clarifications/clade-and-lineage-nomenclature-aids-in-genomic-epidemiology-of-active-hcov-19-viruses/). The Phylogenetic Assignment of Named Global Outbreak Lineages (PANGOLIN) or Pango nomenclature (https://cov-lineages.org/) is one of the most widely used, where newly identified genomes are assigned a lineage based on the global phylogenetic tree (5). The Nextstrain is also an open-source database providing phylogenetic and phylodynamic analysis of SARS-CoV-2 variants grouped into clades based on the year and serial alphabets, 19A, 19B, 20A, 20B, etc. (https://nextstrain.org/ncov/gisaid/global). Additionally, the WHO has assigned Greek alphabets to each lineage, commonly used by the public and media (https://www.who.int/en/activities/tracking-SARS-CoV-2-variants/). For our review, we will be referring to the Pango and/or WHO nomenclatures.

Based on the extensive sequencing data available and observations, the WHO, on conferring with the WHO SARS-CoV-2 Virus Evolution Working Group (https://www.who.int/en/activities/tracking-SARS-CoV-2-variants/) has categorized the SARS-CoV-2 variants that might pose an increased risk to public health into the following three groups:

- **Variants of Concern (VOC)**: VOC is defined by an increase in transmissibility and virulence or decrease in the effectiveness of the practiced public health, social measures and available therapeutics.
- **Variants of Interest (VOI):** VOI is defined by variants observed to cause community spread to appear in multiple cases or clusters or has been detected in various countries.
- **Variants under monitoring (VUM):** VUM is defined as a variant with genetic changes that are suspected to affect virus characteristics with some indication that it may pose a risk to public health and safety in the future. Enhanced monitoring and continuous assessment are required to gather evidence of these variants' phenotypic or epidemiological impacts.

According to the comprehensive data made available by WHO as of December 5, 2021 (https://www.who.int/en/activities/tracking-SARS-CoV-2-variants/), the groups mentioned above are summarized in Table 1. As per critical assessments, including *in-vitro* tests, observed incidence, relative prevalence, etc., the previously designated VOCs and VOIs can be reclassified if they no longer pose a major health risk.

**Table 1:** Summary of VOI and VOC as published by WHO (https://www.who.int/en/activities/tracking-SARS-CoV-2-variants, Last accessed on 17 Dec, 2021).

**WHO label**	**Pango lineage**	**GISAID clade/lineage**	**Status**	**Country of origin**
Alpha	B.1.1.7	GRY (formerly GR/501Y.V1)	VOC:18-Dec-2020	United Kingdom; Sep-2020
Beta	B.1.351	GH/501Y.V2	VOC:18-Dec-2020	South Africa; May-2020
	B.1.351.2			
	B.1.351.3			
Gamma	P.1	GR/501Y.V3	VOC:11-Jan-2021	Brazil; Nov-2020
	P.1.1			
	P.1.2			
Delta	B.1.617.2	G/478K.V1	VOI: 4-Apr-2021 VOC: 11-May-2021	India; Oct-2020
	AY.1			
	AY.2			
Epsilon	B.1.427/B.1.429	GH/452R.V1	VOI:5-Mar-2021	United States of America; Mar-2020
Zeta	P.2	GR/484K.V2	VOI:17-Mar-2021	Brazil; Apr-2020
Eta	B.1.525	G/484K.V3	VOI:17-Mar-2021	Multiple countries; Dec-2020
Theta	P.3	GR/1092K.V1	VOI:24-Mar-2021	Philippines; Jan-2021
Iota	B.1.526	GH/253G.V1	VOI:24-Mar-2021	United States of America; Nov-2020
				Nov-2020
Kappa	B.1.617.1	G/452R.V3	VOI:4-Apr-2021	India; Oct-2020
Lambda	C.37	GR/452Q.V1	VOI:14-06-2021	Peru, Dec-2020
Mu	B.1.621	GH	VOI:30-Aug-2021	Columbia; Jan-2021
Omicron	B.1.1.529	GRA	VUM: 24-Nov-2021	Southern African countries, Nov-2021
			VOC: 26-Nov-2021	

In general, RNA viruses have the highest mutation rates, between 10^−4^ and 10^−6^ mutations per base pair, due to the lack of proofreading ability of RNA-dependent RNA polymerases (RdRp) (6). However, the coronavirus family of viruses are known to have a proofreading mechanism attributed to the exoribonuclease (ExoN) domain of nsp14 (7). The nsp14-ExoN is known to be highly conserved among CoVs (8). Although this was expected to contribute to a low mutation rate, in reality, more than 6 million viral genomes have been recorded over the span of 2 years (GISAID). The first fitness-enhancing mutation on the spike protein was identified just a few months into the evolution of SARS-CoV-2 (9). This could be a result of the sheer magnitude of infection numbers on a global scale. Also, Gribble et al. (8) have experimentally shown that nsp14-ExoN may have a critical role in RNA recombination events during viral replication that can generate genetic variants. In this review, we look into significant mutations observed in viral proteins that are important in the context of diagnostics, therapeutics and vaccine development.

## Variations in SARS-CoV-2 Genome and Proteome

### Spike Protein

The spike glycoprotein is the key protein that defines viral host selection and pathology, and hence the major target for diagnosis and therapy. It is a trimeric transmembrane protein that has two subunits, S1 and S2. The S1 subunit comprises the N-terminal domain (NTD) and the receptor-binding domain (RBD), which is responsible for host-cell attachment. On the other hand, S2 subunit mediates viral entry and contains a fusion peptide (FP) domain, internal fusion peptide (IFP), two heptad-repeat domains (HR1 and HR2), transmembrane domain, and a C-terminal domain (10). Conservation analysis of the SARS-CoV-2 spike protein with other closely related CoVs points at a recombination event that has altered the amino acid sequence of the NTD, RBD and the receptor-binding motif (RBM) sequence within the RBD (11, 12). Specifically, the insertion of a furin cleavage site at the S1/S2 junction, which is absent in SARS-CoV, has shown to contribute to the increased transmissibility (11, 13). The RBD specifically binds to the angiotensin-converting enzyme 2 (ACE2) receptor on the host cell surface, mainly the lung cells and primary human airway epithelial cells (14). The RBD-ACE2 binding causes conformational changes that allow the S2 subunit to mediate the fusion of virus and host cell membranes enabling entry into the host cell (13, 15). Hence, any mutation in the S protein will undoubtedly affect virulence and pathogenicity. Most of the mutations that arise are likely to lessen the virulence or be deleterious. However, the S glycoprotein is the most antigenic viral protein and the major therapeutic target, putting it under constant selective pressure. Therefore, as expected, the S-protein is a mutational hotspot, wherein adaptive mutations may lead to increased transmissibility, infectivity and host immune evasion. Some of the significant mutations in S1 and S2 are described below.

#### S1 Subunit

The S1 is the most immunodominant viral protein, with anti-RBD accounting for almost 90% of the neutralizing antibodies in COVID-19 convalescent plasma. In addition, an antigenic “supersite” was identified in NTD, which is a prominent target for antibody response (9, 16, 17). Interestingly, NTD was found to interact with tyrosine-protein kinase receptor UFO (AXL) highly expressed on pulmonary and bronchial cells, indicating a probable co-receptor involved in viral attachment and entry (18). Considering these facts, frequent mutations in spike S1, especially RBD and NTD, is expected to drive viral adaptation and immune-escape strategy. One of the first identified and highly prevalent mutations is D614G, wherein the aspartic acid at residue 614 (D614) is replaced by glycine (G614). D614G in the RBM domain has shown to increase the S-protein density on the viral surface, thereby enhancing infectivity (19). The D614G substitution is found in most of the circulating VOCs, including Alpha (B.1.1.7), Beta (B.1.351), Delta (B.1.617.2), Gamma (P.1) and the recent delta plus (AY lineage) and Omicron (B.1.1.529) variants (https://www.cdc.gov/coronavirus/2019-ncov/variants/variant-info.html). Some of the other prevalent substitutions in RBD were found to enhance ACE2 binding. Examples include the N501Y, S477N, N439K, D364Y and E484K substitutions identified in most VOCs, which correlate with higher transmissibility (9, 20, 21). The D364Y mutation was found to enhance the spike protein's structural stability, thereby increasing its affinity for the receptor (22). The T478K, Q493K, and Q498R mutations on the recently emerged VOC B1.1.529 (omicron) have shown to double the electrostatic potential, increasing the RBD-ACE2 binding affinity (23). Furthermore, immune evasion arises from conformational modifications due to one or more amino-acid substitutions or deletions. The E484K has also been implicated in immune-escape (20). E484 to K, Q or P was shown to considerably affect convalescent sera neutralization and has been implied in reinfections and vaccine ineffectiveness (9). E484 substitutions have been identified in a number of VOCs, including B.1.351 (E484K), P.1 (E484K), B.1.617.2 (E484K/E484Q) and B.1.1.529 (E484A) (https://covariants.org/variants/S.E484). Similarly, K417N/T found in B.1.351 and P.1 was also found to evade antibody binding, though less potent than E484 substitutions (https://covariants.org/variants/S.E484) (9, 20). Interestingly, the S1-NTD has also harbored a number of mutations, especially deletions, in the course of SARS-CoV-2 evolution. Most of the NTD mutations were found to alter antigenicity or eliminate epitopes, aiding immune-evasion (9, 24). Some of the recurrently deleted regions (RDRs) within the NTD are Δ69–70, Δ141–144, Δ146, Δ210 and Δ243–244 (9). In addition, some of the notable substitutions in NTD are R246I (in B.1.351), W152C (in B.1.429), L18F (in B.1.351 and P.1), T19R (in B.1.17 and B.1.617) and G142D (in B.1.617 lineages), all of which are associated with immune-escape (17, 25, 26).

#### S2 Subunit

The S2 is markedly conserved among CoVs, and have a low mutation rate indicating that most mutations are likely to impact viral entry. Moreover, it is less antigenic, probably due to the extensive N-linked glycosylation, therefore not under much selective pressure compared to S1 (9, 27). However, studies show the HR2 region can elicit an antibody response that is cross-reactive with other CoVs (28). Among the mutations reported, D769H has been described to reduce susceptibility to neutralizing antibodies (9). D950N present on the HR1 domain in Delta lineages have been mapped to the spike trimer interface, suggesting an alteration in spike dynamics for the highly virulent delta strain (25). Unlike the previous VOCs, the B.1.1.529 (Omicron variant) surprisingly exhibit a number of S2 substitutions, namely D796Y, N856K, Q954H, N969K and L981F (https://covariants.org/variants/21K.Omicron). The steady spread of B.1.1.529 across the globe suggests a positive advantage for these mutations. However, the impact of these mutations on viral pathogenicity and polyclonal mAb response (vaccine-induced and convalescent) is yet to be determined.

Typically, a cluster of mutations is selected during evolution that can act synergistically, providing a broader adaptive advantage. The delta variants carry the L452R and T478K mutations in the RBD, and E156del–F157del in the N-terminus that are implicated in immune escape. Additionally, P681R mutation at the S1–S2 cleavage site is thought to increase viral replication, leading to higher viral loads and increased transmission (29). The omicron variant possesses >32 mutations on the spike protein, including five on the RBD, making it highly divergent from the original Wuhan strain, indicating a high chance of immune escape. Moreover, it shares several mutations with other VOCs, along with newly identified substitutions. Table 2 summarizes the prominent spike mutations in currently circulating VOCs.

**Table 2:** Mutations in the spike protein of SARS-CoV-2 variants that may be contributing to increased pathological properties.

**Sl. no:**	**Mutation**	**Region on spike**	**VOCs**	**Impact on viral pathogenicity**
1	D614G	RBD	Found in several lineages	Appeared in 2020 and is the most prevalent. Increase spike density (19)
2	N501Y	RBD	B.1.1.7, B.1.351, P.1	Antibody escape (9)
				May effect host tropism (30)
3	E484K/Q/A	RBD	B.1.351, P.1, B.1.617.1, B.1.1.529	Increase ACE2 binding
				Antibody escape—vaccine ineffectiveness and reinfections (9)
5	K417N/T	RBD	B.1.351, P.1,	Antibody escape—vaccine ineffectiveness and reinfections (9)
6	L452R, T478K	RBD	B.1.617	Increase ACE2 binding (31, 32)
				Antibody escape—resistance to antibody drugs (33)
7	Q677P/H	Near 1/S2 cleavage	Found in several lineages B.1.525	May play a role in increasing the penetrability of the virus into human cells. Not yet shown to be highly infectious.
8	T478K, Q493K, and Q498R	RBD	B.1.1.529	Predicted to increase RBD-ACE2 binding (34)
9	Δ69-70	NTD	B.1.1.7, B.1.1.529	Immune escape (9)

Yet, it is also important to note that immune response against SARS-CoV-2 comprises humoral (neutralizing antibodies produced by B-cells) and cellular response (T-cells). Most studies consider only the IgG response against spike epitopes. Increasing data suggest that variants with antibody-escape spike mutations do not evade T-cell response (35, 36).

### M and E Proteins

Both M and E proteins are highly conserved with low mutation rates and therefore serve as important screening markers for coronavirus infection. M-protein is the most abundant and is responsible for maintaining the shape of the virion by spanning the membrane bilayer. The M-protein also facilitates the budding of the viral particles from the host cells, and enhances glucose transport in the host cell during viral replication. It has a sequence similarity of 98% to bat and pangolin CoV M proteins (37). Interestingly, the M-protein was found to elicit IgM response during the acute phase of SARS-CoV-2 infection (38). A number of mutations have been reported in the M-protein. Shen et al. (39) report the increased prevalence of I82T and V70L mutations in several lineages, suggesting it is beneficial for the virus, probably by facilitating increased glucose uptake. Bianchi et al. (37) predict that mutations occurring at the N-terminal region of M-protein may play a key role in host-cell interaction. The common mutations detected in the N-terminal domain are V5F, E8D, V5I, and Y2H, which might affect the viral efficiency (40). The implications of these mutations in IgM response and pathogenicity remains to be elucidated. Similarly, the E-protein is also conserved in nature and bears sequence similarity to pangolin and bat CoV E-proteins (37). The E-protein is a hydropathic transmembrane protein, rich in valine and leucine residues, that plays a role in the pathogenesis of the virus. Overall, the M and E genes exhibit fewer mutations than the S-protein (41). Comprehensive mutational analysis from GISAID database reports <2% E-mutant strains (42). However, higher amino acid variations in the C-terminal domain of the E-protein, such as S55F, V62F and R69I, may affect the binding of SARS-Cov-2 E-protein to the tight junction proteins impacting pathogenesis (42).

### N Protein

The nucleocapsid is an important viral protein/gene with respect to diagnostics (nucleic acid and antigen detection) and new vaccine design (43, 44). Its function is to maintain the genome structure inside the envelope and plays significant roles in viral assembly, budding, and the host cellular response to viral infection (45). The N gene is highly conserved among CoVs and is more stable with lower mutation rates than the S-protein (46–48). In addition, the N-protein has been identified as an important target for T-cell response, making it a suitable candidate for next-generation COVID-19 vaccines against emerging variants (44, 49, 50). *In-silico* studies show that mutations in the N-terminal domain of N protein affect the structure and flexibility of the protein, whereas substitutions in the C-termini are believed to impact the dimerization potential (51). The common mutations observed in N-protein are R203K and G204R (52). However, the impact of N-protein mutations on infectiousness and transmission rate is yet to be determined. For instance, it was found that the European variant 20A.EU1 carrying N-mutation A220V and the S-mutation A222V had become dominant in summer 2020, probably an outcome of synergistic effect (53). The omicron harbors a comparatively high number of deletions in the N-gene, which has been reported to impact diagnostics, mainly primer binding of a few commercially approved kits. The impact of these mutations on viral pathogenicity is yet to be elucidated.

### Non-structural Proteins

The ORF1a/b gene is an important target for the nucleic acid diagnostics of SARS-CoV-2. It codes for non-structural proteins (nsp1-16), responsible for the replication machinery and maintenance of the viral genome (54). Some of these nsp proteins are targets for anti-viral drugs that are currently used for COVID-19 treatment, such as RdRp (nsp12) and 3-chyomotrypsin like protease (3CLpro, nsp5 also known as main protease or M^pro^) and papain-like proteinase protein (PL^pro^, nsp3). Adaptive mutations in ORF1a/b may lead to increased viral replication or drug resistance, thus enhancing virulence. An early geographical distribution study of ORF1a/b mutations report maximum mutation rate for RdRp (33.47%), followed by nsp2 protein (20.04%), nsp13 helicase (15.95%) and nsp3 proteins (12.61%). Mutations on the other nsp proteins ranged between 0.14% for nsp10 and 2.79% for nsp6 (55). The RdRp is the key replication enzyme, and is expected to be well conserved to preserve functionality. Nevertheless, studies have reported point mutations in ORF1a/b corresponding to RdRp, which are P4715L, P323L and T265I b (56). Both P4715L and P323L was observed along with S protein D614G mutation, suggesting a co-evolutionary pattern (56, 57). Studies also describe that deletion at amino-acid 500–532 in the nsp1 gene is associated with retained ribosomal binding ability, higher RT-PCR cycle thresholds, and lower serum IFN-β levels of infected patients (52). Variations in the other nsp genes have also been described. Fitness-enhancing mutations have been reported in nsp3, namely F206F, S1197R and T1198K, that has been associated with increased severity of infection for B.1.1 lineages (52). Further, certain mutations in 3CLpro: T45I, K90R, R279C, A266V, A234Vand N151D have been found in the VOCs B.1.1.7, B.1.351, P.1 and B1.617 (58). Mohammad et al. (58) further speculate T190I and A191V in 3CLpro can alter polarity and affect the binding of therapeutic molecules. Moreover, several geographical studies have been published, where Indian samples reveal a high mutation frequency in nsp2, nsp3, nsp4, nsp5, nsp6, nsp12, nsp14, and nsp16 (59). Whereas Wang et al. (57) report frequent mutations in nsp2 and nsp13 in the US.

### Other ORFs and Accessory Proteins

Mutations in accessory proteins is most likely to impact the function of the protein negatively. Keeping in mind the viability, most of these proteins are likely to be conserved. Nonetheless, specific prevalent mutations have been observed. ORF3 and ORF8 have been noted as mutational hotspots, whereas ORF6, ORF7a, ORF7b, ORF10 have been observed to be conserved with lower mutation rates (56). In the US, Q57H mutation in the ORF3a region, and S24L and L84S in ORF8 was found to be prevalent, suggesting a positive effect on transmission or virulence (60, 61).

**Figure 2:**
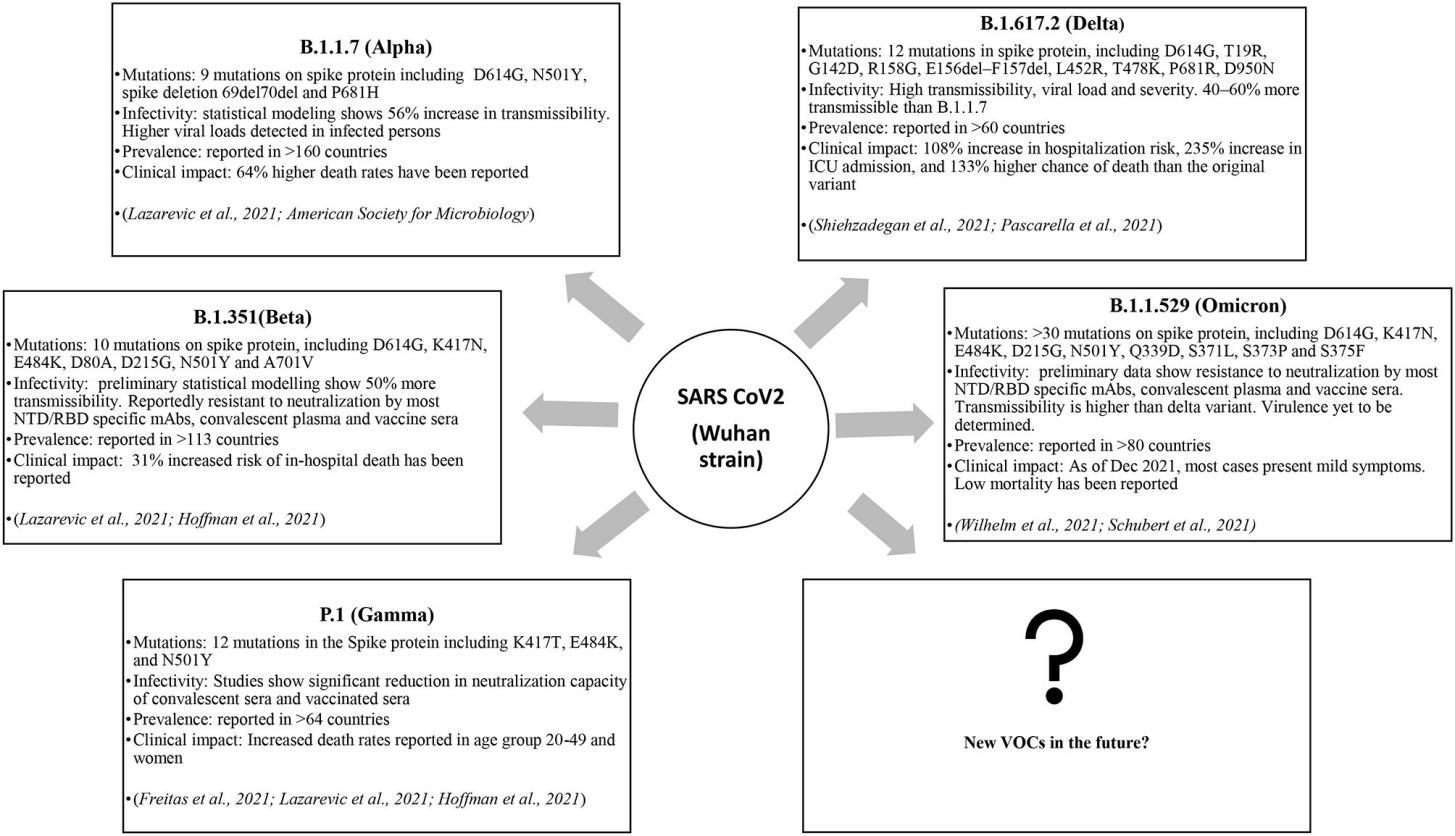
The VOCs and their impact.

Thus, the mutational spectra of the SARS-CoV-2 (Table 3) is a crucial factor to be taken into account while using current diagnostic assays, targeted therapeutics and vaccines, and also for designing new armamentarium against the emergence of potentially virulent variants. In this regard, continued efforts are imperative to monitor the impact of mutations on currently used therapeutics and diagnostics and further track the genomic variability between individuals and across geographical areas. Figure 2 depicts the epidemiological characteristics of the currently circulating VOCs.

**Table 3:** Key defining mutations for VOCs (https://covariants.org/).

		**B.1.1.7 (Alpha)**	**B.1.351 (Beta)**	**P.1 (Gamma)**	**B.1.617.2 (Delta)**	**B.1.1.529 (Omicron)**
Mutations on structural proteins	Spike	H69del, V70del, Y144del, N501Y, A570D, D614G, P681H, T716I, S982A, D1118H	D80A, D215G, L241del, L242del, A243del, K417N, E484K, N501Y, D614G, A701V	L18F, T20N, P26S, D138Y, R190S, K417T, E484K, N501Y, D614G, H655Y, T1027I, V1176F,	T19R, E156del, F157del, R158G, L452R, T478K, D614G, P681R, D950N	A67V, H69del, V70del, T95I, G142del, V143del, Y144del, Y145D, N211del, L212I, G339D, S371L, S373P, S375F, K417N, N440K, G446S, S477N, T478K, E484A, Q493R, G496S, Q498R, N501Y, Y505H, T547K, D614G, H655Y, N679K, P681H, N764K, D796Y, N856K, Q954H, N969K, L981F
	Nucleocapsid	D3L, R203K, G204R, S235F	T205I	P80R, R203K, G204R	D63G, R203M, D377Y	P13L, E31del, R32del, S33del, R203K, G204R
	Envelope		P71L			T9I
	Membrane				I82T	D3G, Q19E, A63T
Mutations on non-structural proteins	ORF1a	T1001I, A1708D, I2230T, S3675del, G3676del, F3677del	T265I, K1655N, K3353R, S3675del, G3676del, F3677del	S1188L, K1795Q, S3675del, G3676del, F3677del		K856R, S2083del, L2084I, A2710T, T3255I, P3395H, L3674del, S3675del, G3676del, I3758V
	ORF1b	P314L	P314L	P314L, E1264D	P314L, G662S, P1000L	P314L, I1566V
	ORF3a		Q57H	S253P	S26L	
	ORF7a				V82A, T120I	
	ORF8	Q27^*^, R52I, Y73C		E92K	D119del, F120del	
	ORF9b				T60A	P10S, E27del, N28del, A29del

* *Stop codon*.

## Impact on Diagnostics

The current molecular *in-vitro* diagnostic landscape for COVID-19 is either based on: (i) genomic detection assays, (ii) serological tests for antibody detection, and (iii) viral antigen detection.

### Nucleic Acid Tests

The two main technology platforms used for genomic detection of SARS-CoV-2 are real-time reverse transcription polymerase chain reaction (rRT-PCR) and high throughput sequencing. Other point-of-care PCR techniques such as Loop-mediated isothermal amplification (LAMP-PCR) have also been developed. The RT-PCR is considered the gold standard for SARS-CoV-2 detection from patient nasopharyngeal samples. Generally, multiple genes are targeted in a diagnostic assay to ensure specificity and sensitivity to SARS-CoV-2. Some of the viral genes that are used for diagnostics include S, N (N1 and N2 regions within the N gene), E, RdRp (nsp12), nsp14, etc. (62). The spike protein has nucleotide sequences unique to SARS-CoV-2, therefore minimizing cross-reactivity and false-positive results (FPRs) in the presence of other CoVs. However, since the S-gene harbors frequent mutations, commercial kits and probe sets must be regularly validated to detect new variants to avoid false-negative results (FNRs). S-gene target failure (SGTF) has been reported in the case of Alpha (63) and Omicron variants for TaqPath™ kit from Thermo Fisher Scientific, which also targets ORF1ab and N Protein. The 69-70del mutation on S causes an S-gene dropout among the three targets, and this phenomenon is currently being used for surveillance of the newly emerged omicron variant (https://www.thermofisher.com/) (64). Although the other genes such as N and RdRp are less prone to mutations, any mutation in the primer binding region can reduce assay sensitivity and result in FNRs. An analytical study by Rahman et al. (42) describes mutations within the N-gene that could affect the sensitivity of RT-PCR tests. For instance, Vanaerschot et al. (65) reports an SNP Q289H in N-gene impacted forward primer binding and markedly reduced RT-PCR assay sensitivity. Furthermore, the US FDA has identified a few commercially approved test kits that could be negatively impacted by the prevalent SARS-CoV-2 variants (FDA) (66). The Revogene SARS-CoV-2 (Meridian Bioscience, Inc.) and DTPM COVID-19 RT-PCR (Tide Laboratories, LLC), both single target tests that detects N-gene, is expected to produce FNR for omicron variants as it harbors nine-nucleotide deletion in the N-gene, spanning positions 28370-28362 (66). Similarly, Linea COVID-19 Assay Kit (Applied DNA Sciences, Inc.) targets two S-gene epitopes which have been mutated in omicron. As a proxy, S and N-gene drop out assays are being used for the early distinction of omicron (S–/N–) and delta (S+/N+) variants.

### Viral Antigen Tests

These tests detect viral proteins in patient blood or nasopharyngeal samples using specific antibodies. Antigen tests are ideal point-of-care tests for screening suspected or random population at public places, including airports and hospital-in patients, or as self-tests. They are mainly enzyme-linked immunosorbent assay (ELISAs) or lateral flow assays (LFAs) that detect epitopes of viral antigens specific to SARS-CoV-2, usually the—N or N+S protein. In the scenario of a rapidly evolving virus, monoclonal antibody assays targeting a single epitope can have low sensitivity and test accuracy rates. Hence, polyclonal antibodies to multiple epitopes can be considered a better and more feasible option. Studies by Ascoli (67) propose polyclonal anti-N antibodies to be sensitive against the N501Y, H69/V70, D796H and D614G mutations. Some commercial kits that detect N-gene, such as SARS-CoV-2 Rapid Antigen Test (Roche), Panbio COVID-19 Ag RAPID (Abott) and CLINITEST Rapid COVID-19 (Siemens Healthcare), were found to be valid for the VOCs B.1.1.7 (Alpha), B.1.351 (Beta), P.1 (Gamma), and B.1.617.2 (Delta) (68). The N-mutations in the B.1.1.529 (Omicron) may affect the accuracy of some of the approved commercial antigen tests.

### Antibody Tests

These tests detect serum antibodies in patients formed as an immune response to the SARS-CoV-2. In the context of a global vaccination drive, serological tests hold importance in studying sero-prevalence and vaccine effectiveness. Out of all the viral antigens, the S-protein and N-protein display the highest immunogenicity. The anti-N antibodies appear first during infection (69), followed by the more dominant anti-S antibodies (70). Therefore, most serological tests detect S or N directed antibodies in patient blood samples using ELISA, LFA or immunofluorescence. Moreover, IgM and IG titer levels represent the early or convalescent phase of infection, respectively. With respect to mutations and emergence of new serotypes, changes in secondary or tertiary structures of the protein used in the assay can impact the test as the patient antibodies might not recognize the new structure. Hence, the use of multiple fragments of S and N proteins will improve accuracy against variants. However, to date no rigorous studies have been performed to evaluate the impact of variants on the analytic or clinical sensitivity of approved antibody tests.

To summarize, RT-PCR remains the most appropriate diagnostic method to test infection positivity, and serological tests to detect vaccine effectiveness. A number of the commercially available diagnostic assays are likely to be impacted by the omicron variant. It remains critical to continuously monitor serotypes and evaluate diagnostic kits to detect new and emerging variants as and when reported. Moreover, regional and country-wise surveillance is also important considering the geographical dissimilarity in the prevalence of different variants.

## Impact on Therapeutics

The first line of COVID-19 drugs in clinical use is broad-spectrum antivirals such as nucleotide analogs (example, Remdesivir, Favipiravir) or glucose analogs (2-deoxy-d-glucose) that are usually not impacted by mutations. Moreover, anti-inflammatory drugs to suppress cytokine storm in patients with severe conditions are also unaffected by mutations. However, several drugs are under development, and mutations can have repercussions in targeted therapies using small-molecule drugs, biologics or convalescent plasma.

### Small Molecule Drugs

With the help of bioinformatics tools, a wide range of small molecule drugs are being screened to target various viral enzymes involved in host-cell entry or replication (2). Some important drug targets include the Spike-RBD region, RdRp, nucleocapsid and nsp 5 (3CLpro) (71). Nucleotide substitutions and deletions can alter the polarity and secondary structures of viral proteins. Consequently, it can interfere with the binding to small molecule drugs. The major class of antiviral small-molecule drugs that are in use against SARS-CoV-2 are RdRp inhibiting nucleoside analogs that work by introducing mutations in the viral RNA or by halting replication. However, the presence of nsp14-ExoN activity in SARS-CoV-2 can limit the action of these drugs (72). The new-generation RdRp inhibitors such as Remdesivir and Favipiravir have improved analog chemistry resistant to ExoN activity. Early studies had identified mutations on RdRp and nsp14 genes (24, 73). However, their correlation to viral pathogenicity, higher mutation load, or drug-resistance needs to be further investigated. To date, there is no data available comparing the effectiveness of Remdesivir against variants, although *in-silico* studies show that mutations such as F480L, V557L, D722Y, V472D and L469S on RdRp may disrupt the binding capacity of Remdesivir (71). Further investigation revealed Alanine at 156th of RdRp is critical for drug binding, implying any substitutions in this position would affect efficacy (51). Similarly, the effectiveness of lopinavir/ritonavir (that target 3CL^Pro^) against variants are not reported yet. Several novel anti-viral therapeutics are under development. A recent *in-silico* study suggests Conivaptan, Ergotamine, Venetoclax and Rifapentine as promising target for N-protein, which are mostly conserved across variants (51).

### Monoclonal Antibodies

Humanized or fully-human engineered therapeutic antibodies have high specificity and mimic natural antibodies produced by the immune system. Numerous neutralizing monoclonal antibodies (mAbs) are currently under development to combat COVID-19, a gamut of them targeting the spike protein (4). As of December 2021, the US FDA has approved four mAb therapies for COVID-19.

- **Casirivimab-imdevimab mAb cocktail** targeting two different epitopes of S-protein. Both the mAbs together were found to be active against most of the circulating variants. However, *in-vitro* neutralization assays show that casirivimab alone (without imdevimab) had reduced activity against (i) K417N+ E484K substitutions found in P.1 lineage (Brazil variant), and (ii) E484Q mutation expressed in B.1.617.1/B.1.617.3 lineages (Delta/Indian variants) (FDA) (74). In addition, the casirivimab was shown to be moderately neutralizing and imdevimab non-neutralizing toward the B.1.1.529 (omicron) that carries a high number of mutations in the spike (75). Specific mutations in the RBD of omicron, namely T478K, Q493K, Q498R, and E484A are thought to impact mAb binding efficacy (23). Another study by Wilheim et al. (75), state the mAb cocktail is effective against delta variants but failed against omicron.
- **Bamlanivimab and Etesevimab mAb cocktail** targeting two different epitopes of S-protein RBD. According to US FDA reports, the mAb cocktail retained neutralization activity against B.1.1.7 (carrying N501Y) and B.1.617.2/AY.3 (carrying L452R + T478K). However, it showed a significant reduction in the neutralization of B.1.351 (carrying K417N + E484K +N501Y). For the P.1 variant carrying K417T + E484K + N501Y, neutralization assays using pseudotyped virus-like particles showed a reduction in neutralization (FDA) (76).
- **Sotrovimab** targeting an S-protein epitope. *In-vitro* assays confirm that the drug retains neutralization activity against most of the VOCs that are currently prevalent, including B.1.617, B.1.351, P.1 and B.1.1.7. However, it has been warned that if P337H/L/R/T and E340A/K/G arises, the mAb can show a reduced susceptibility of more than 100-fold (FDA) (77).
- **Tixagevimab and cilgavimab** antibodies are derived from B-cells donated by COVID-19 convalescent sera. The mAbs target spike RBD epitopes that are quite close to each other, possibly interacting with each other but essentially not competing for binding (33). *In-vitro* analysis on chimeric viruses showed slightly reduced potency of tixagevimab against B.1.351 (with K417N + E484K + N501Y) and P.1 (with K417N + E484K + N501Y). And cilgavimab had lower activity against N501Y+D614G mutants, including B.1.429 (carrying L452R), B.1.617.2 (carrying L452R + T478K) and B.1.351 (carrying K417N + E484K + N501Y) (33). However, the authors report no significant impact on the combined efficacy of maAbs on VOCs B.1.1.7, B.1.351 or P.1 (33). Further, Shah and Woo report cilgavimab to be moderately neutralizing against the B.1.1.529 (omicron variant), wheras Tixagevimab showed a marked drop (23).

Since most of these tests were done on pseudotype virus-like particles with selected spike mutations, the actual neutralization potential against the different viral strains and newly emerging mutants is yet to be determined. Wang et al. (60, 61), reported that the P.1 variant is completely resistant to casirivimab, bamlanivimab and etesevimab. Furthermore, the Center for Disease Control and Prevention (CDC), USA, warns health professionals of potential reduction of clinical efficacy toward several circulating strains (CDC) (78). In such a case, identification of viral strain before mAb administration in patients will be necessary, which is a non-feasible task. In the setting of a fast-mutating pandemic virus, a single monoclonal antibody treatment might not be the best choice for therapeutics. The chances of escape mutations for a specific antibody are always higher. Therefore, polyclonal antibodies, cocktail antibodies or multivalent or multispecific antibodies (e.g., bispecifics) are a better strategy. In this regard, several new therapeutic antibody formats, including bispecifics, antibody-fragments and nanobodies, are under development.

### Convalescent Plasma and Post-vaccination Sera

Like mAb therapies, plasma therapies derived from recovered patients and vaccinated individuals have been affected by viral variants. Hence, it implies the possibility of reinfection and vaccine ineffectiveness. Compared to mAb therapies, plasma therapies may have a broader neutralization activity due to polyclonal antibodies. But even then, several variants have been indicated as potentially resistant to convalescent plasma and post-vaccinated sera therapies (CDC) (78). According to the CDC, US, the P.1 variant seems more resistant to plasma therapy than B.1.351, B.1.617.2, B.1.427, B.1.429 and B1.1.7. The antibody evasion of many variants has been attributed to E484K mutation on the spike protein (60, 61, 79). More details about the sensitivity of variants to post-vaccine sera are discussed in the upcoming section.

## Impact on Vaccines

The rising mortality and infection rates expedited vaccine development across the globe. The five common types of platforms utilized to generate covid-19 vaccines are (i) Live-attenuated or inactivated vaccine, (ii) Protein subunit, (iii) Viral vector, (iv) Nucleic acid vaccine (mRNA and plasmid DNA), and (v) virus-like particle vaccine (80, 81). As mentioned earlier, RNA viruses exhibit higher mutation rates than DNA viruses (82, 83) and therefore, mutations in the S-gene (84, 85), the target for leading mRNA and adeno-viral vector vaccines, may impact the reactivity with neutralizing antibodies. Here, we primarily focus on the vaccines, presently licensed for use in various countries, to understand their efficacy on the currently prevailing VOCs B.1.1.7, B.1.351, P.1, B.1.617.2 and B.1.1.529 (summarized in Table 4).

**Table 4:** Impact of current VOCs on the neutralizing efficacy of vaccines.

	** 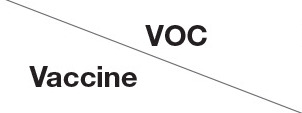 **	**B.1.1.7 (Alpha)**	**B.1.351 (Beta)**	**P.1 (Gamma)**	**B.1.617.2 (Delta)**	**B.1.1.529 (Omicron)**	**References**
RNA based platform	Pfizer-BioNTech Vaccine	✓	Neutralization efficacy slightly reduced	✓	Neutralization efficacy slightly reduced	Poor neutralizing efficacy	(61, 86, 87)
	Moderna vaccine	✓	Neutralization efficacy slightly reduced	Neutralization efficacy slightly reduced	Neutralization efficacy slightly reduced	Poor neutralizing efficacy	(61, 87)
Adenoviral Vector	Astra-Zeneca/Oxford vaccine (Covishield or Vaxzevria)	✓	Neutralization efficacy slightly reduced	✓	✓	Poor neutralizing efficacy	(86–90)
	Gamelaya institute—Sputnik V	✓	Neutralization efficacy slightly reduced	✓	✓	Unclear	(91, 92)]
	Janssen vaccine	✓	✓	✓	✓	Poor neutralizing efficacy	(87, 93)
Inactivated virus	Sinovac—CoronaVac	✓	Unclear	✓	Unclear	Unclear	WHO
	Sinopharm BIBP vaccine	✓	✓	✓	✓	Unclear	WHO
	Bharat Biotech—Covaxin	✓	✓	✓	✓	Unclear	(94, 95) (bharatbiotech.com)
Protein-Based	Novavax/Covovax (NVX-CoV2373)	✓	Neutralization efficacy slightly reduced	✓	✓	Unclear	(96)

### MRNA Vaccines

mRNA vaccines deliver the genetic code of the target protein in the mRNA form, encapsulated in an absorbable lipid structure (97). The advantage of mRNA vaccines with respect to new emerging variants is the ability to easily modify the mRNA sequence of the target in case of any significant mutations, and the relatively quick manufacturability.

The currently approved mRNA vaccines are Pfizer (BNT162b2) and Moderna (mRNA-1273), both of which encode a spike protein ectodomain of the Wuhan strain (98). Several *in-vitro* neutralization assays using pseudo-virus systems were tested to determine the cross-neutralizing ability of post-vaccine sera. In the case of B.1.1.7, studies by Xiu et al. and Wu et al. did not report a significant impact in the neutralizing capacity of sera of humans immunized with Pfizer and Moderna, respectively (99, 100). Similarly, experiments by Wang et al. (60, 61) demonstrated modest reductions in the sera from individuals that received Moderna or Pfizer vaccines for the B.1.1.7 (1.8–2-fold). Whereas, concerning B.1.351, sera derived from individuals vaccinated with Pzifer or Moderna exhibited reductions by 6.5-fold or 8.6-fold, respectively, in a study conducted by Wang et al. (60, 61). Moreover, a prominent reduction was observed in the neutralization of B.1.351 by sera from either humans or non-human primates (NHPs) who received Moderna vaccine (99). Likewise, to understand the impact of the Moderna vaccine against P.1, a small laboratory study by Dejnirattisai et al. (101) observed antibody titers to reduce by 2.6-fold. Although this effect is yet to be confirmed in clinical trials. Likewise, to comprehend the efficacy of Pfizer vaccine against P.1, Liu and colleagues conducted a small laboratory study using vaccine sera which suggests roughly equivalent neutralization of this variant (102). However, experiments by Dejnirattisai et al. and Parry et al. suggest that antibody titers were reduced by 2.6-fold and 14-fold, respectively. Albeit the impact is yet to be confirmed in clinical trials (101, 103). Furthermore, a report released from Public Health England has stated that the Pfizer-BioNTech is 87.9% effective against symptomatic disease caused by the B.1.617.2 variant 2 weeks following the second dose (29). Neutralization studies also showed 5.8 fold reduction in spike binding for Pfizer vaccine (86). Although the two vaccines showed a drop in neutralization, studies point out they are still effective against severe symptoms and hospitalization (104). The emergence of omicron has raised many concerns regarding mRNA vaccine efficacy owing to high mutations on the spike. Preliminary experiments using sera from Pfizer-BioNTech-vaccinated individuals indicate a substantial reduction in neutralization potency against B.1.1.529 (75).

### Non-replicating Viral Vector Vaccines

Non-replicating viral vector vaccines generally use an engineered adenovirus to deliver the DNA code for the target protein. All of the currently approved adenoviral vector vaccines for SARS-CoV-2 target the spike protein. A major disadvantage with adenoviral vaccines is the chances of the vaccinated individual developing immunity against the adenovirus (97). In such cases, a second dose or revaccinations with the same platform for new variants could be challenging.

Studies on the Oxford-AstraZeneca vaccine by Emary et al. and Kunal et al. reported an overall reduced neutralization activity, but retention of efficacy against B.1.1.7 variant of SARS-CoV-2 compared to the non-B.1.1.7 variants *in-vitro* (88, 105). However, Oxford/AstraZeneca COVID-19 vaccine (AZD1222) failed to protect against mild-to-moderate COVID-19 infection due to the B.1.351 variant (89, 90). Whereas, in both P.1 variant and B.1.617.2 variant, the vaccine did retain its efficacy (105). Bernal et al. (29), reported 59.8% effectiveness against symptomatic disease caused by the B.1.617.2 variant 2 weeks following the second dose of the Oxford-AstraZeneca vaccine. Further, *in-vitro* tests show a 2.5 fold decrease in neutralization for B.1.617.2 using post-vaccinated sera (after 2 doses) (86). Initial reports also state a reduced neutralizing potency for the vaccine against B.1.1.529 (87).

In the case of CanSinoBio (Convidecia), as per the report released by Pakistan authorities, initially, the vaccine demonstrated 65.7% efficacy in preventing symptomatic cases and a 90.98% success rate in stopping severe disease in an interim analysis of global trials. But no reports have been yet released with regards to the prevailing variants (106).

The WHO-approved Janssen vaccine (Ad26.COV2.S) has been tested against a variety of SARS-CoV-2 virus variants in clinical trials, including B1.351, P.1 and B.1.617.2 and found to be efficacious as reported on their website (www.jnj.com). Jongeneelen et al. (93) report a 3.6-fold, 3.4-fold and 1.6-fold decrease in post-vaccine sera neutralization against B.1.351, P.1 and B.1.617.2, respectively. However, the vaccine showed a significant reduction in binding toward B1.1.529 RBD (87).

Sputnik V(Gam-COVID-Vac) developed by the Gamalaya Research Institute is a two-vector vaccine (Ad26+Ad25) that has been approved in several countries. The vaccine has demonstrated ~three-fold decrease in neutralization activity for B.1.351, 2-fold for P.1 and 2.5-fold for B.1.617.2 (92).

### Inactivated Virus Vaccines

Inactivated virus is the most traditional and time-tested vaccination strategy. The major downsides of the inactivated virus are the extensive manufacturing timelines and low immunogenicity. These types of vaccines generally require of high dose of antigen for a significant immune response. However, inactivated vaccines exhibit a polyclonal antibody response against multiple viral antigens, including spike, nucleocapsid and other proteins. In the circumstances of emerging variants, this attribute has the edge over other vaccine platforms. Therefore, in the dynamic scenario of the pandemic, an inactivated virus must be able to elicit a high antibody titer in order to have a strong cross-neutralizing potency.

Regarding the CoronaVac (Sinovac R&D Company) vaccine, sera collected from vaccinated individuals were found effective against B.1.1.7, similar to the effectiveness elicited against the wildtype strain. Whereas, the efficiency significantly reduced in the case of P.1, and only a small proportion of post-vaccine sera exhibited neutralization against B.1.351 variants (107). BBV152/COVAXIN is another inactivated vaccine that was recently authorized by the WHO. The vaccine demonstrated a 71% efficacy against all variant-related COVID-19 illnesses, with 90% efficacy against Kappa and 65% against Delta (94, 95).

### Protein Subunit Vaccine

A protein subunit vaccine usually contains a protein or a polysaccharide unit of the infectious agent. An advantage of this platform is that the manufacturing platform uses the well-established recombinant technology that is widely available. Also, the transport and storage does not require −20°C or −80°C cold chains, therefore global distribution can take place at regular refrigeration temperatures (108).

The first protein subunit vaccine approved for COVID-19 is the Novavax candidate NVX-CoV2373, a recombinant nanoparticle protein-based vaccine. It uses the full-length spike protein organized around a nanoparticle core and formulated with the proprietary Matrix-M adjuvant (108). Ideally, this design should enable a strong polyclonal antibody production against various epitopes, including cryptic/hidden epitopes. In clinical trials, NVX-CoV2373 has shown 89.7% effectiveness against symptomatic Covid-19 caused by both B.1.1.7 and non-B.1.1.7 variants during late 2020. However, this vaccine has shown only 51% efficacy against the B.1.351 variant (96).

## Discussion and Future Directions

The main reason the SARS-CoV-2 virus spread to pandemic proportions is the presence of asymptomatic and mild infections or an infection phase that can go undetected. Thus, it becomes complicated to trace, track and control movements of infected persons, making them carriers and super-spreaders. With such high infection rates, the emergence and spread of mutants were inevitable. Although vaccines have been developed at a record pace to fight the pandemic, the emergence of SARS-CoV-2 variants that can evade vaccine immunity would cause new waves of infections. RNA viruses are also known to exist as quasispecies populations, and as a result of the ongoing vaccination and therapeutic efforts, the virus is under increasing selective pressure (109). Experiments show that selective pressure from monoclonal antibodies leads to quicker escape mutations. In comparison, polyclonal antibodies from post-vaccine sera and convalescent plasma are more likely to induce a delayed emergence of escape mutations (110).

**Figure 3:**
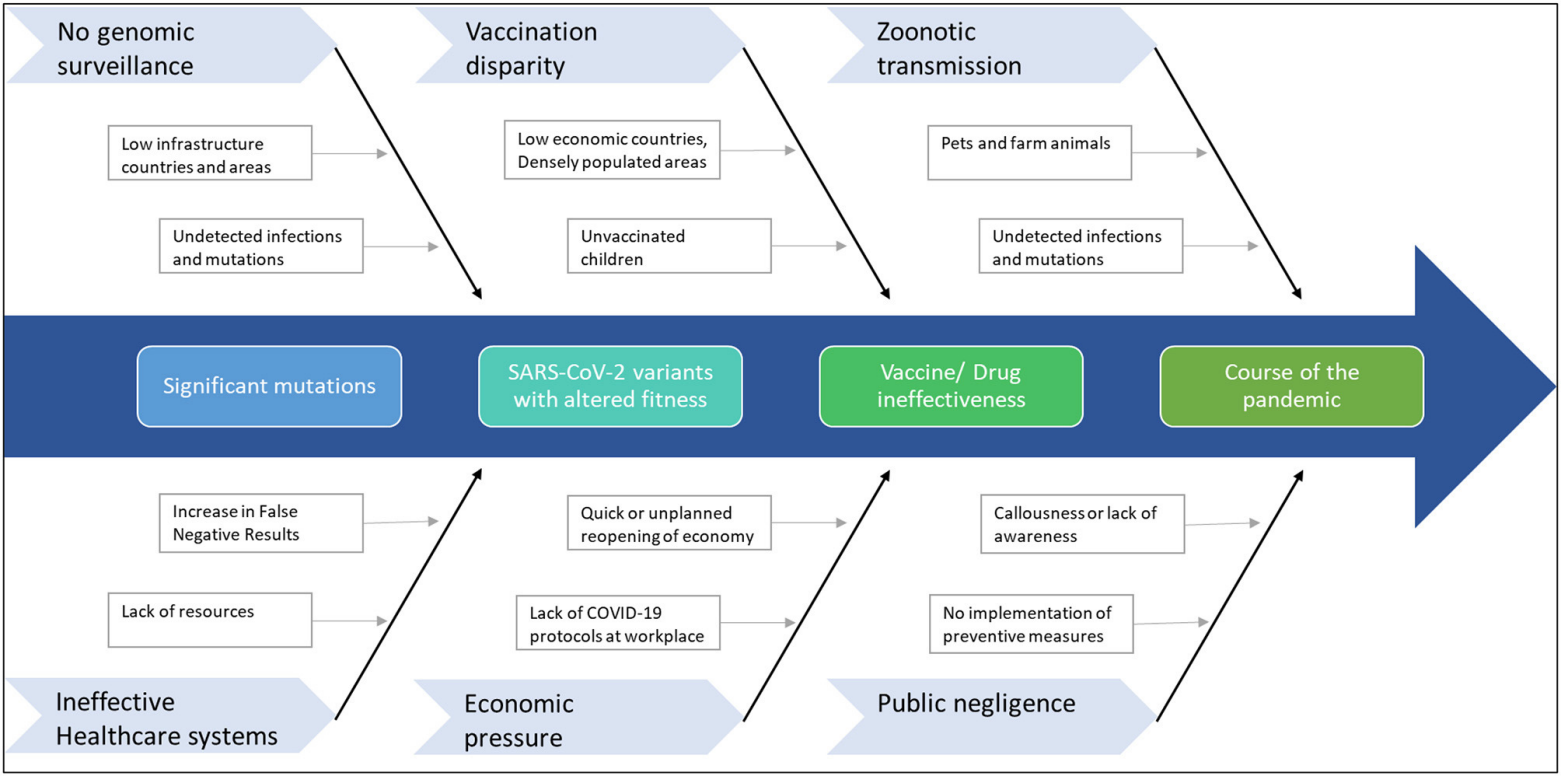
Factors that can contribute to further mutations in SARS-CoV-2. The emergence of any immune-escape variants can change the course of the pandemic.

Given the scenario, response strategies to tackle the rise of further mutants that can extend the pandemic is of paramount importance (Figure 3). Therefore, primarily as part of the road map, continuous genomic surveillance for mutations concerning geographical variations is an absolute necessity. Subsequently, global and government efforts are required to increase testing and sequencing capacity. Low economic zones and countries with limited resources trail behind the quest for genomic sampling and vaccinations. Such consequences facilitate blind spots for the evolution of numerous strains, which can again spread far and wide. In addition, unvaccinated children and pets or farm animals also pose a considerable risk of churning out mutants. In the long run, such circumstances can cause multiple pandemic waves and raise the demand for vaccine redesign. Although the concept of redesigning mRNA and adenoviral vector vaccines seems relatively straightforward, the redesigning of vaccines is burdensome apropos to manufacturing and clinical testing to approve the new product.

As per the reports, the currently approved vaccines broadly show cross-neutralization ability for variants. However, a reduction in *in-vitro* neutralization efficacy has been observed with some vaccines concerning VOCs and specific mutations. As a result, a higher incidence of breakthrough infections may be expected for the currently prevalent delta and the surging omicron. Moreover, a number of studies report waning of vaccine-induced immunity after 6–7 months. For the Pfizer-BioNTech vaccine, Naaber et al. (111) reported that after 6 months the anti-RBD IgG levels plunged to 2–25% from their peak levels detected after the second dose. Likewise, Levine-Tiefenbrun et al. (112) describe a marked decline in the protective effect of Pfizer-BioNTech vaccine after 6 months. Further, Khoury et al. outline a predictive model for vaccine-induced immunity. They estimate that the neutralization level required for protection from severe disease infection is ~six-fold lower than the level required for protection against symptomatic/mild infection. Therefore it can be said that even with waning antibody levels, there will be protection against severe disease (113). Another very important aspect that needs to be factored in is vaccine-induced T-cell immunity. Reassuringly, studies show the mutations in VOCs (alpha, beta, gamma and delta) do not impact the T-cell response elicited by vaccines or natural infection (110, 114). Unlike antibody immunity generated against exposed epitopes, T-cell immunity acts against multiple viral proteins, including unexposed epitopes. In addition, Zuo et al. (115) report that T-cell response is maintained at six months from primary infection.

Taken together, these findings indicate a requirement for timely booster vaccines. However, with the arrival of omicron, another question arises: is this the right time to implement vaccine redesigns? What could be the strategies for next-generation COVID-19 vaccines? Interestingly, the conception of cocktail vaccines has been proposed as a more effective strategy in the context of a rapidly mutating virus (116). The idea is to use a combination of structural and non-structural viral antigens for a broad-range immunity. Furthermore, the nucleocapsid structural protein, a more conserved gene with good immunogenicity, has also been proposed as an ideal vaccine candidate to combat SARS-CoV-2 variants (46, 117). Also, bioinformatic studies on conserved epitopes of the N protein suggest nucleocapsid vaccines can provide cross-reactive immune protection against multiple human coronaviruses (47, 118). In addition, the N-protein is a major target for T-cell response that will be able to protect against severe symptoms and emerging variants (44). Another important criterion to combat variants is to ensure that the vaccines induce high neutralization titers. Sera with high neutralization titers (convalescent and post-vaccination) were found to be more effective in conferring protection against variants (79). Most of the focus lately has been on developing chimeric vaccines and vaccines that induce a more durable and broad-spectrum T-cell immunity. This has indeed shifted the priority from S1-RBD to S1 coupled with S2 and/or N (44, 119–121). Nevertheless, mutations that can evade T-cell response can also arise, which calls for active surveillance (122).

**Figure 4:**
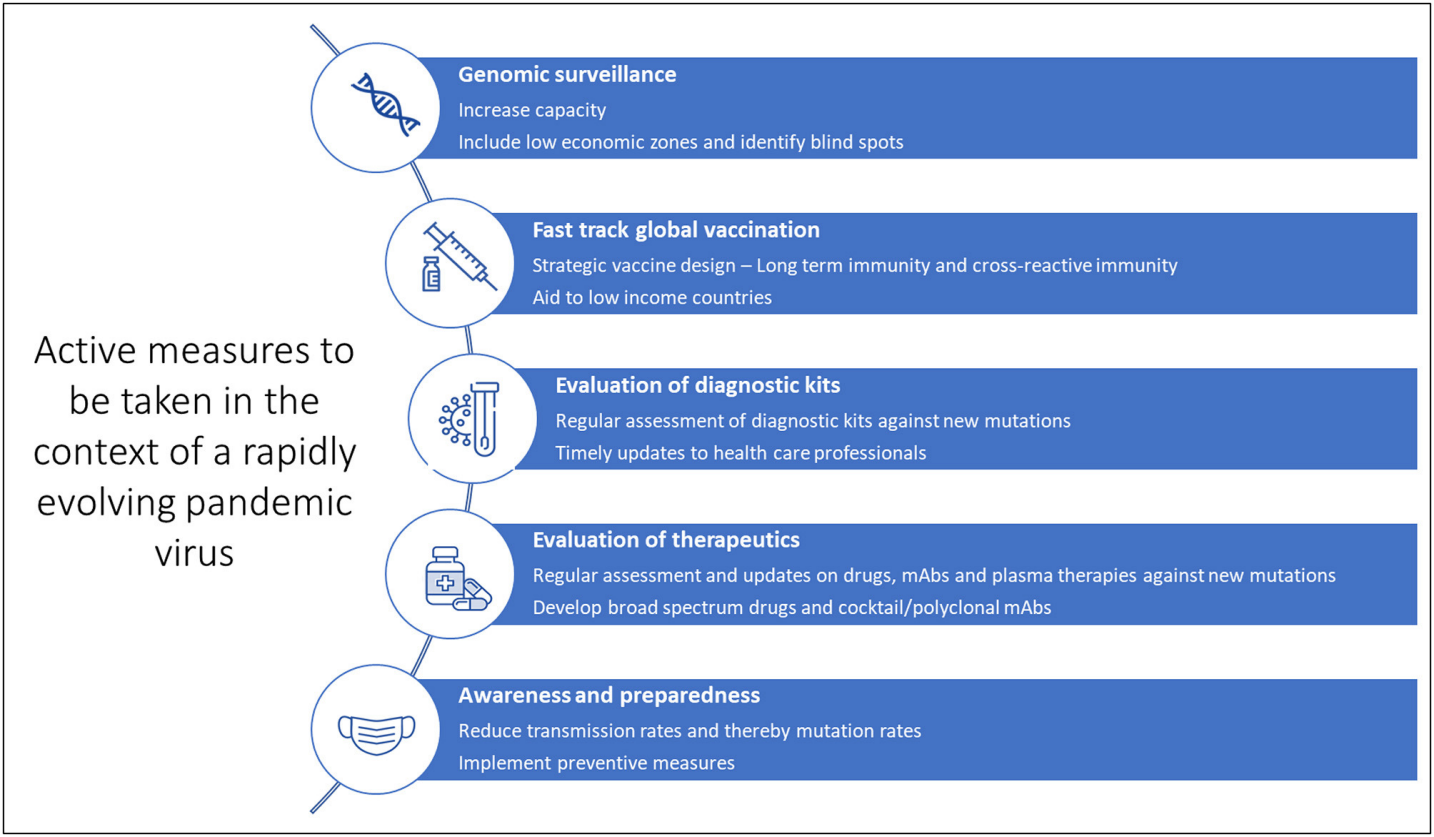
Active measures against the evolving SARS-CoV-2.

While we may have to wait several months for the next-generation of more potent vaccines to tackle the emergence of dangerous variants, it is important to continue vaccination programs and administration of booster doses. Although reduced effectiveness may be observed, the first-generation vaccines still protect against severe disease. To conclude, continued collaboration between the scientific community, healthcare systems, administration systems and the general public will help curb the pandemic (Figure 4). General awareness and active implementation of preventive measures at individual and population levels are crucial. Health care providers and clinical laboratory personnel must be regularly updated on mutations and their impact on diagnostics, therapeutics and vaccines for timely and appropriate medical management. And last but not least, spreading awareness and educating the general public is critical at such uncertain times. As always, prevention is indeed better than cure.

## Author Contributions

SS, SPi, SPh, AN, and DS prepared the draft contents. RS, ST, and GV reviewed and finalized the article. All authors contributed to the article and approved the submitted version.

## Funding

Seed funding was received from the kind support of Andhra Pradesh Medtech Zone and other support from in-house funding of Trivitron Healthcare.

## Conflict of Interest

SS was employed as contract scientific writer by the company Blue Horizon International Therapeutic Sciences, USA. AN was employed by MP Advisors. ST, SPh, and GV are from Trivitron Healthcare Pvt., Ltd. The remaining authors declare that the research was conducted in the absence of any commercial or financial relationships that could be construed as a potential conflict of interest.

## Publisher's Note

All claims expressed in this article are solely those of the authors and do not necessarily represent those of their affiliated organizations, or those of the publisher, the editors and the reviewers. Any product that may be evaluated in this article, or claim that may be made by its manufacturer, is not guaranteed or endorsed by the publisher.

## References

[1] Chattu VK Kumar R Kumary S Kajal F David JK. Nipah virus epidemic in southern India and emphasizing “One Health” approach to ensure global health security. J Fam Med Primary Care. (2018) 7:275–83. 10.4103/jfmpc.jfmpc_137_18 30090764 PMC6060941

[2] Majumder J Minko TJTAJ. Recent developments on therapeutic and diagnostic approaches for COVID-19. AAPS J. (2021) 23:1–22. 10.1208/s12248-020-00532-2 33400058 PMC7784226

[3] Mohamadian M Chiti H Shoghli A Biglari S Parsamanesh N Esmaeilzadeh AJTJ. COVID-19: virology, biology and novel laboratory diagnosis. J Gene Med. (2021) 23:e3303. 10.1002/jgm.3303 33305456 PMC7883242

[4] Wang M-Y Zhao R Gao L-J Gao X-F Wang D-P Cao J-M. SARS-CoV-2: structure, biology, and structure-based therapeutics development. Front. Cell. Infect. Microbiol. (2020) 10:724. 10.3389/fcimb.2020.587269 33324574 PMC7723891

[5] Rambaut A Holmes EC O'Toole Á Hill V McCrone JT Ruis C. A dynamic nomenclature proposal for SARS-CoV-2 lineages to assist genomic epidemiology. Nat Microbiol. (2020) 5:1403–7. 10.1038/s41564-020-0770-5 32669681 PMC7610519

[6] Sanjuán R Nebot MR Chirico N Mansky LM Belshaw R. Viral mutation rates. J Virol. (2010) 84:9733–48. 10.1128/JVI.00694-10 20660197 PMC2937809

[7] Robson F Khan KS Le TK Paris C Demirbag S Barfuss P. *Coronavirus RNA*. proofreading: molecular basis and therapeutic targeting. Mol Cell. (2020) 79:710–27. 10.1016/j.molcel.2020.11.048 32853546 PMC7402271

[8] Gribble J Stevens LJ Agostini ML Anderson-Daniels J Chappell JD Lu X. The coronavirus proofreading exoribonuclease mediates extensive viral recombination. PLoS Pathog. (2021) 17:e1009226. 10.1371/journal.ppat.1009226 33465137 PMC7846108

[9] Harvey WT Carabelli AM Jackson B Gupta RK Thomson EC Harrison EM. SARS-CoV-2 variants, spike mutations and immune escape. Nat Rev Microbiol. (2021) 19:409–24. 10.1038/s41579-021-00573-0 34075212 PMC8167834

[10] Xia X. Domains and functions of spike protein in sars-cov-2 in the context of vaccine design. Viruses. (2021) 13:109. 10.3390/v13010109 33466921 PMC7829931

[11] Johnson J. Positive New Data for Johnson Johnson Single-Shot COVID-19 Vaccine on Activity Against Delta Variant Long-lasting Durability of Response. (2021). Available online at: https://www.jnj.com/positive-new-data-for-johnson-johnson-single-shot-covid-19-vaccine-on-activity-against-delta-variant-and-long-lasting-durability-of-response (accessed December 27, 2021).

[12] Lei KC Zhang XD. Conservation analysis of SARS-CoV-2 spike suggests complicated viral adaptation history from bat to human. Evol Med Public Health. (2020) 2020:290–303. 10.1093/emph/eoaa041 33372198 PMC7665476

[13] Peacock TP Goldhill DH Zhou J Baillon L Frise R Swann OC. The furin cleavage site in the SARS-CoV-2 spike protein is required for transmission in ferrets. Nat Microbiol. 181:1–11. 10.1038/s41564-021-00908-w 33907312 PMC7619196

[14] Yang Y Du L. SARS-CoV-2 spike protein: a key target for eliciting persistent neutralizing antibodies. Signal Transduct Target Ther. (2021) 6:95. 10.1038/s41392-021-00523-5 33637679 PMC7908000

[15] Juraszek J Rutten L Blokland S Bouchier P Voorzaat R Ritschel T. Stabilizing the closed SARS-CoV-2 spike trimer. Nat Commun. (2021) 12:244. 10.1038/s41467-020-20321-x 33431842 PMC7801441

[16] Gupta D Sharma P Singh M Kumar M Ethayathulla A Kaur P. Structural and functional insights into the spike protein mutations of emerging SARS-CoV-2 variants. Cell Mol Life Sci. (2021) 78:7967–89. 10.1007/s00018-021-04008-0 34731254 PMC11073194

[17] McCallum M De Marco A Lempp FA Tortorici MA Pinto D Walls AC. N-terminal domain antigenic mapping reveals a site of vulnerability for SARS-CoV-2. Cell. (2021) 184:2332–47.e2316. 10.1016/j.cell.2021.03.028 33761326 PMC7962585

[18] Wang S Qiu Z Hou Y Deng X Xu W Zheng T. AXL is a candidate receptor for SARS-CoV-2 that promotes infection of pulmonary and bronchial epithelial cells. Cell Res. (2021) 31:126–40. 10.1038/s41422-020-00460-y 33420426 PMC7791157

[19] Zhang L Jackson CB Mou H Ojha A Peng H Quinlan BD. SARS-CoV-2 spike-protein D614G mutation increases virion spike density and infectivity. Nat Commun. (2020) 11:6013. 10.1038/s41467-020-19808-4 33243994 PMC7693302

[20] Barton MI MacGowan SA Kutuzov MA Dushek O Barton GJ van der Merwe PA. Effects of common mutations in the SARS-CoV-2 Spike RBD and its ligand, the human ACE2 receptor on binding affinity and kinetics. Elife. (2021) 10:e70658. 10.7554/eLife.70658.sa2 34435953 PMC8480977

[21] Liu Y Liu J Plante KS Plante JA Xie X Zhang X. The N501Y spike substitution enhances SARS-CoV-2 transmission. bioRxiv. (2021). 10.1101/2021.03.08.434499 [Online ahead of print]. 34818667 PMC8900207

[22] Chen J Wang R Wang M Wei GW. Mutations strengthened SARS-CoV-2 infectivity. J Mol Biol. (2020) 432:5212–26. 10.1016/j.jmb.2020.07.009 32710986 PMC7375973

[23] Woo HG Shah M. Omicron: a heavily mutated SARS-CoV-2 variant exhibits stronger binding to ACE2 and potently escape approved COVID-19 therapeutic antibodies. Front Immunol. (2021). 10.3389/fimmu.2021.830527 35140714 PMC8819067

[24] Khateeb J Li Y Zhang H. Emerging SARS-CoV-2 variants of concern and potential intervention approaches. Critical Care. (2021) 25:1–8. 10.1186/s13054-021-03662-x 34253247 PMC8274962

[25] Planas D Veyer D Baidaliuk A Staropoli I Guivel-Benhassine F Rajah MM. Reduced sensitivity of SARS-CoV-2 variant Delta to antibody neutralization. Nature. (2021) 596:276–80. 10.1038/s41586-021-03777-9 34237773

[26] Shen L Triche TJ Bard JD Biegel JA Judkins AR Gai X. Spike Protein NTD mutation G142D in SARS-CoV-2 Delta VOC lineages is associated with frequent back mutations, increased viral loads, and immune evasion. medRxiv. (2021). 10.1101/2021.09.12.21263475

[27] Barrett CT Neal HE Edmonds K Moncman CL Thompson R Branttie JM. Effect of mutations in the SARS-CoV-2 spike protein on protein stability, cleavage, and cell-cell fusion function. bioRxiv. (2021). 10.1101/2021.01.24.428007 34157282 PMC8214756

[28] Ladner JT Henson SN Boyle AS Engelbrektson AL Fink ZW Rahee F. Epitope-resolved profiling of the SARS-CoV-2 antibody response identifies cross-reactivity with endemic human coronaviruses. Cell Rep Med. (2021) 2:100189. 10.1016/j.xcrm.2020.100189 33495758 PMC7816965

[29] Bernal JL Andrews N Gower C Gallagher E Simmons R Thelwall S. Effectiveness of Covid-19 Vaccines against the B.1.617.2 (Delta) Variant. J Med. (2021). 385:585–594. 10.1056/NEJMoa2108891 34289274 PMC8314739

[30] Richard M Kok A de Meulder D Bestebroer TM Lamers MM Okba NMA. SARS-CoV-2 is transmitted via contact and via the air between ferrets. Nat Commun. (2020) 11:3496. 10.1038/s41467-020-17367-2 32641684 PMC7343828

[31] Giacomo SD Mercatelli D Rakhimov A Giorgi FM. Preliminary report on severe acute respiratory syndrome coronavirus 2 (SARS-CoV-2) Spike mutation T478K. J Med Virol. (2021) 93:5638–43. 10.1002/jmv.27062 33951211 PMC8242375

[32] Tchesnokova V Kulasekara H Larson L Bowers V Rechkina E Kisiela D. Acquisition of the L452R mutation in the ACE2-binding interface of spike protein triggers recent massive expansion of SARS-CoV-2 variants. J Clin Microbiol. (2021) 59:e0092121. 10.1128/JCM.00921-21 34379531 PMC8525575

[33] Dong J Zost S Greaney A Starr TN Dingens AS Chen EC. Genetic and structural basis for recognition of SARS-CoV-2 spike protein by a two-antibody cocktail. Nat Microbiol. (2021). 6:1233–44 (2021). 10.1038/s41564-021-00972-2 34548634 PMC8543371

[34] Shah M Woo HG. Omicron: A heavily mutated SARS-CoV-2 variant exhibits stronger binding to ACE2 and potently escape approved COVID-19 therapeutic antibodies. BioRxiv [Preprint]. (2021). 10.1101/2021.12.04.471200 35140714 PMC8819067

[35] Geers D Shamier MC Bogers S den Hartog G Gommers L Nieuwkoop NN. SARS-CoV-2 variants of concern partially escape humoral but not T cell responses in COVID-19 convalescent donors and vaccine recipients. Sci Immunol. (2021) 6:eabj. 10.1126/sciimmunol.abj1750 34035118 PMC9268159

[36] Mahajan S Kode V Bhojak K Karunakaran C Lee K Manoharan M. Immunodominant T-cell epitopes from the SARS-CoV-2 spike antigen reveal robust pre-existing T-cell immunity in unexposed individuals. Sci Rep. (2021) 11:13164. 10.1038/s41598-021-92521-4 34162945 PMC8222233

[37] Bianchi M Benvenuto D Giovanetti M Angeletti S Ciccozzi M Pascarella SJBRI. Sars-CoV-2 envelope and membrane proteins: structural differences linked to virus characteristics? Biomed Res Int. (2020) 2020:1–6. 10.1155/2020/4389089 32596311 PMC7261327

[38] Jörrißen P Schütz P Weiand M Vollenberg R Schrempf IM Ochs K. Antibody response to SARS-CoV-2 membrane protein in patients of the acute and convalescent phase of COVID-19. Front Immunol. (2021) 12:679841. 10.3389/fimmu.2021.679841 34421894 PMC8371319

[39] Shen L Bard JD Triche TJ Judkins AR Biegel JA Gai XJE. Emerging variants of concern in SARS-CoV-2 membrane protein: a highly conserved target with potential pathological and therapeutic implications. Emerg Microb Infect. (2021) 10:885–93. 10.1080/22221751.2021.1922097 33896413 PMC8118436

[40] Mou K Abdalla M Wei DQ Khan MT Lodhi MS Darwish DB. Emerging mutations in envelope protein of SARS-CoV-2 and their effect on thermodynamic properties. Inform Med Unlocked. (2021) 25:100675. 10.1016/j.imu.2021.100675 34337139 PMC8314890

[41] Jakhmola S Indari O Kashyap D Varshney N Das A Manivannan E. Mutational analysis of structural proteins of SARS-CoV-2. Heliyon. (2021) 7:e06572. 10.1016/j.heliyon.2021.e06572 33778179 PMC7980187

[42] Rahman MS Hoque MN Islam MR Islam I Mishu ID Rahaman MM. Mutational insights into the envelope protein of SARS-CoV-2. Gene Rep. (2021) 22:100997. 10.1016/j.genrep.2020.100997 33319124 PMC7723457

[43] Diao B Wen K Zhang J Chen J Han C Chen Y. Accuracy of a nucleocapsid protein antigen rapid test in the diagnosis of SARS-CoV-2 infection. Clin Microbiol Infect. (2021) 27:289.e281–4. 10.1016/j.cmi.2020.09.057 33031947 PMC7534827

[44] Matchett WE Joag V Stolley JM Shephard FK Quarnstrom CF Mickelson CK. Nucleocapsid vaccine elicits spike-independent SARS-CoV-2 protective immunity. bioRxiv. (2021). 10.1101/2021.04.26.441518 34193597 PMC8516699

[45] Gao T Gao Y Liu X Nie Z Sun H Lin K. Identification and functional analysis of the SARS-COV-2 nucleocapsid protein. BMC Microbiol. (2021) 21:58. 10.1186/s12866-021-02107-3 33618668 PMC7898026

[46] Dutta NK Mazumdar K Gordy JT. The nucleocapsid protein of SARS-CoV-2: a target for vaccine development. J Virol. (2020) 94:00647–20. 10.1128/JVI.00647-20 32546606 PMC7307180

[47] Oliveira SC de Magalhães MT Homan EJJF. Immunoinformatic analysis of SARS-CoV-2 Nucleocapsid protein and identification of COVID-19 vaccine targets. Front Immunol. (2020) 11:2758. 10.3389/fimmu.2020.587615 33193414 PMC7655779

[48] Rahman MS Islam MR Alam ARU Islam I Hoque MN Akter S. Evolutionary dynamics of SARS-CoV-2 nucleocapsid protein and its consequences. J Med Virol. (2021) 93:2177–95. 10.1002/jmv.26626 33095454

[49] Dangi T Class J Palacio N Richner JM MacMaster PP. Combining spike-and nucleocapsid-based vaccines improves distal control of SARS-CoV-2. Cell Rep. (2021) 36:109664. 10.1016/j.celrep.2021.109664 34450033 PMC8367759

[50] Le Bert N Tan AT Kunasegaran K Tham CYL Hafezi M Chia A. SARS-CoV-2-specific T cell immunity in cases of COVID-19 and SARS, and uninfected controls. Nature. (2020) 584:457–62. 10.1038/s41586-020-2550-z 32668444

[51] Azad GK. The molecular assessment of SARS-CoV-2 nucleocapsid phosphoprotein variants among Indian isolates. Heliyon. (2021) 7:e06167. 10.1016/j.heliyon.2021.e06167 33553784 PMC7848562

[52] Ramesh S Govindarajulu M Parise RS Neel L Shankar T Patel S. Emerging SARS-CoV-2 variants: a review of its mutations, its implications and vaccine efficacy. Vaccines. (2021) 9:1195. 10.3390/vaccines9101195 34696303 PMC8537675

[53] Vilar S Isom DGJB. One year of SARS-CoV-2: How much has the virus changed? Biology. (2021) 10:91. 10.3390/biology10020091 33530355 PMC7911924

[54] Mishra SK Tripathi TJA. One year update on the COVID-19 pandemic: Where are we now? Actatropica. (2020) 214:105778. 10.1016/j.actatropica.2020.105778 33253656 PMC7695590

[55] Guruprasad K. Geographical distribution of amino acid mutations in human SARS-CoV-2 orf1ab poly-proteins compared to the equivalent reference proteins from China. ChemRxiv. (2021). 10.33774/chemrxiv-2021-lf2zd-v2

[56] Omotoso OE Babalola AD Matareek A. Mutational hotspots and conserved domains of SARS-CoV-2 genome in African population. Beni-Suef Univ J Basic Appl Sci. (2021) 10:1–7. 10.1186/s43088-021-00102-1 33564691 PMC7861160

[57] Wang R Chen J Gao K Hozumi Y Yin C Wei GW. Analysis of SARS-CoV-2 mutations in the United States suggests presence of four substrains and novel variants. Commun Biol. (2021) 4:228. 10.1038/s42003-021-01754-6 33589648 PMC7884689

[58] Mohammad T Choudhury A Habib I Asrani P Mathur Y Umair M. Genomic variations in the structural proteins of SARS-CoV-2 and their deleterious impact on pathogenesis: a comparative genomics approach. Front Cell Infect Microbiol. (2021) 11:765039. 10.3389/fcimb.2021.765039 34722346 PMC8548870

[59] Biswas N Kumar K Mallick P Das S Kamal IM Bose S. Structural and drug screening analysis of the non-structural proteins of severe acute respiratory syndrome coronavirus 2 virus extracted from Indian coronavirus disease 2019 patients. Front Genet. (2021) 12:747–751. 10.3389/fgene.2021.626642 33767730 PMC7985531

[60] Wang P Casner RG Nair MS Wang M Yu J Cerutti G. Increased resistance of SARS-CoV-2 variant P.1 to antibody neutralization. Cell Host Microbe. (2021) 29:747–51.e744. 10.1016/j.chom.2021.04.007 33887205 PMC8053237

[61] Wang P Nair MS Liu L Iketani S Luo Y Guo Y. Antibody resistance of SARS-CoV-2 variants B.1.351 and B.1.1.7. Nature. (2021) 593:130–5. 10.1038/s41586-021-03398-2 33684923

[62] Wang R Hozumi Y Yin C Wei GW. Mutations on COVID-19 diagnostic targets. Genomics. (2020) 112:5204–13. 10.1016/j.ygeno.2020.09.028 32966857 PMC7502284

[63] American Society for Microbiology,. B.1.1.7: What We Know About the Novel SARS-CoV-2 Variant. (2021). Available online at: https://asm.org/Articles/2021/January/B-1-1-7-What-We-Know-About-the-Novel-SARS-CoV-2-Va (accessed November 14, 2021).

[64] ThermoFisher. The S Gene Advantage: TaqPath COVID-19 Tests May Help with Early Identification of Omicron Variant. (2021). ThermoFisher

[65] Vanaerschot M Mann SA Webber JT Kamm J Bell SM Bell J. Identification of a polymorphism in the N gene of SARS-CoV-2 that adversely impacts detection by reverse transcription-PCR. J Clin Microbiol. (2020) 59:e02369–e02320. 10.1128/JCM.02369-20 33067272 PMC7771441

[66] US FDA,. SARS-CoV-2 Viral Mutations: Impact on COVID-19 Tests. (2021). Available online at: https://www.fda.gov/medical-devices/coronavirus-covid-19-and-medical-devices/sars-cov-2-viral-mutations-impact-covid-19-tests (accessed July 6, 2021).

[67] Ascoli CA. Could mutations of SARS-CoV-2 suppress diagnostic detection? Nat Biotechnol. (2021) 39:274–5. 10.1038/s41587-021-00845-3 33603204

[68] Jungnick S Hobmaier B Mautner L Hoyos M Haase M Baiker A. In vitro rapid antigen test performance with the SARS-CoV-2 variants of concern B 11 7 (Alpha), B 1351 (Beta), P 1 (Gamma), and B 1617 2 (Delta). Microorganisms. (2021) 9:1967. 10.3390/microorganisms9091967 34576862 PMC8465346

[69] Smits VA Hernández-Carralero E Paz-Cabrera MC Cabrera E Hernández-Reyes Y Hernández-Fernaud JR. The Nucleocapsid protein triggers the main humoral immune response in COVID-19 patients. Biochem Biophys Res Commun. (2021) 543:45–9. 10.1016/j.bbrc.2021.01.073 33515911 PMC7825866

[70] Huang AT Garcia-Carreras B Hitchings MDT Yang B Katzelnick LC Rattigan SM. A systematic review of antibody mediated immunity to coronaviruses: kinetics, correlates of protection, and association with severity. Nat Commun. (2020) 11:4704. 10.1038/s41467-020-18450-4 32943637 PMC7499300

[71] Zeng L Li D Tong W Shi T Ning B. Biochemical features and mutations of key proteins in SARS-CoV-2 and their impacts on RNA therapeutics. Biochem Pharmacol. (2021) 189:114424. 10.1016/j.bcp.2021.114424 33482149 PMC7816569

[72] Khater S Kumar P Dasgupta N Das G Ray S Prakash A. Combining SARS-CoV-2 proofreading exonuclease and RNA-dependent RNA polymerase inhibitors as a strategy to combat COVID-19: a high-throughput in silico screening. Front Microbiol. (2021) 12:647693. 10.3389/fmicb.2021.647693 34354677 PMC8329495

[73] Eskier D Suner A Oktay Y Karakülah G. Mutations of SARS-CoV-2 nsp14 exhibit strong association with increased genome-wide mutation load. PeerJ. (2020) 8:e10181. 10.7717/peerj.10181 33083157 PMC7560320

[74] US FDA,. Fact Sheet for Health Care Providers Emergency Use Authorization (EUA) of REGEN-COV (Casirivimab With Imdevimab). (2021). Available online at: https://www.fda.gov/media/145611/download (accessed July 5, 2021).

[75] Wilhelm A Widera M Grikscheit K Toptan T Schenk B Pallas C. Reduced neutralization of SARS-CoV-2 omicron variant by vaccine sera and monoclonal antibodies. medRxiv. (2021). 10.1101/2021.12.07.2126743234818119

[76] US FDA,. Fact Sheet for Health Care Providers Emergency Use Authorization (EUA) of Bamlanivimab Etesevimab. (2021). Available online at: https://www.fda.gov/media/145802/download (accessed July 5, 2021).

[77] US FDA,. Fact Sheet for Health Care Providers Emergency Use Authorization (EUA) of Sotrovimab. (2021). Available online at: https://www.fda.gov/media/149534/download (accessed July 5, 2021).

[78] CDC CDCP,. SARS-CoV-2 Variant Classifications Definitions. (2021). Available online at: https://www.cdc.gov/coronavirus/2019-ncov/variants/variant-info.html (accessed July 5, 2021).

[79] Jangra S Ye C Rathnasinghe R Stadlbauer D Alshammary H Amoako AA. SARS-CoV-2 spike E484K mutation reduces antibody neutralisation. Lancet Microbe. (2021) 2:e283–e284. 10.1016/S2666-5247(21)00068-9 33846703 PMC8026167

[80] Kyriakidis NC Lopez-Cortes A Gonzalez EV Grimaldos AB Prado EO. SARS-CoV-2 vaccines strategies: a comprehensive review of phase 3 candidates. NPJ Vaccines. (2021) 6:28. 10.1038/s41541-021-00292-w 33619260 PMC7900244

[81] Nagy A Alhatlani B. An overview of current COVID-19 vaccine platforms. Comput Struct Biotechnol J. (2021) 19:2508–17. 10.1016/j.csbj.2021.04.061 33936564 PMC8076774

[82] Duffy S. Why are RNA virus mutation rates so damn high? PLoS Biol. (2018) 16:e3000003. 10.1371/journal.pbio.3000003 30102691 PMC6107253

[83] Lauring AS Andino R. Quasispecies theory and the behavior of RNA viruses. PLoS Pathog. (2010) 6:e1001005. 10.1371/journal.ppat.1001005 20661479 PMC2908548

[84] Li Q Wu J Nie J Zhang L Hao H Liu S. The impact of mutations in SARS-CoV-2 spike on viral infectivity and antigenicity. Cell. (2020) 182:1284–94e1289. 10.1016/j.cell.2020.07.012 32730807 PMC7366990

[85] van Dorp L Acman M Richard D Shaw LP Ford CE Ormond L. Emergence of genomic diversity and recurrent mutations in SARS-CoV-2. Infect Genet Evol J Mol Epidemiol Evol Genet Infect Dis. (2020) 83:104351. 10.1016/j.meegid.2020.104351 32387564 PMC7199730

[86] Bian L Gao Q Gao F Wang Q He Q Wu X. Impact of the Delta variant on vaccine efficacy and response strategies. Expert Rev Vaccines. (2021) 20:1201–9. 10.1080/14760584.2021.1976153 34488546 PMC8442750

[87] Schubert M Bertoglio F Steinke S Heine PA Ynga-Durand MA Zuo F. Human serum from SARS-CoV-2 vaccinated and COVID-19 patients shows reduced binding to the RBD of SARS-CoV-2 Omicron variant in comparison to the original Wuhan strain and the Beta and Delta variants. medRxiv. (2021). 10.1101/2021.12.10.21267523 PMC8890955 35236358

[88] Emary KRW Golubchik T Aley PK Ariani CV Angus B Bibi S. Efficacy of ChAdOx1 nCoV-19 (AZD1222) vaccine against SARS-CoV-2 variant of concern 202012/01 (B. 1.1. 7): an exploratory analysis of a randomised controlled trial. Lancet. (2021) 397:1351–62. 10.1016/S0140-6736(21)00628-0 33798499 PMC8009612

[89] Gomez CE Perdiguero B Esteban M. Emerging SARS-CoV-2 variants and impact in global vaccination programs against SARS-CoV-2/COVID-19. Vaccines. (2021) 9:9030243. 10.3390/vaccines9030243 33799505 PMC7999234

[90] Madhi SA Baillie V Cutland CL Voysey M Koen AL Fairlie L. Efficacy of the ChAdOx1 nCoV-19 Covid-19 vaccine against the B. 1.351 variant. New Engl J Med. (2021) 384:1885–98. 10.1056/NEJMoa2102214 33725432 PMC7993410

[91] Ikegame S Siddiquey M Hung CT Haas G Brambilla L Oguntuyo KY. (2021). Neutralizing activity of Sputnik V vaccine sera against SARS-CoV-2 variants. MedRxiv [Preprint] (2021). 10.1101/2021.03.31.21254660 34312390 PMC8313705

[92] Gushchin VA Dolzhikova IV Shchetinin AM Odintsova AS Siniavin AE Nikiforova MA. Neutralizing activity of sera from Sputnik V-vaccinated people against variants of concern (VOC: B. 1.1. 7, B. 1.351, P. 1, B. 1.617. 2, B. 1.617. 3) and Moscow endemic SARS-CoV-2 variants. Vaccines. (2021) 9:779. 10.3390/vaccines9070779 34358195 PMC8310330

[93] Jongeneelen M Kaszas K Veldman D Huizingh J van der Vlugt R Schouten T. Ad26. COV2. S elicited neutralizing activity against Delta and other SARS-CoV-2 variants of concern. bioRxiv. (2021). 10.1101/2021.07.01.450707

[94] Sapkal G Yadav PD Ella R Abraham P Patil DY Gupta N. Neutralization of VUI B. 1.1. 28 P2 variant with sera of COVID-19 recovered cases and recipients of Covaxin an inactivated COVID-19 vaccine. J Travel Med. (2021) 28:taab077. 10.1093/jtm/taab077 34002240 PMC8194512

[95] Sapkal GN Yadav PD Ella R Deshpande GR Sahay RR Gupta N. Inactivated COVID-19 vaccine BBV152/COVAXIN effectively neutralizes recently emerged B. 1.1. 7 variant of SARS-CoV-2. J Travel Med. (2021) 28:taab051. 10.1093/jtm/taab051 33772577 PMC8083765

[96] Heath PT Galiza EP Baxter DN Boffito M Browne D Burns F. Safety and efficacy of NVX-CoV2373 Covid-19 vaccine. N Engl J Med. (2021) 385:1172–83. 10.1056/NEJMoa2107659 34192426 PMC8262625

[97] Welsh J. Coronavirus variants-will new mRNA vaccines meet the challenge? Engineering. (2021) 7:712–4. 10.1016/j.eng.2021.04.005 33898075 PMC8053359

[98] Bordon YJNRI. Variant constraint by mRNA vaccines. Nat Rev Immunol. (2021) 21:274–5. 10.1038/s41577-021-00548-5 33837367 PMC8034506

[99] Wu K Werner AP Moliva JI Koch M Choi A Stewart-Jones GBE. mRNA-1273 vaccine induces neutralizing antibodies against spike mutants from global SARS-CoV-2 variants. bioRxiv. (2021). 10.1101/2021.01.25.427948 33501442 PMC7836112

[100] Xie X Liu Y Liu J Zhang X Zou J Fontes-Garfias CR. Neutralization of SARS-CoV-2 spike 69/70 deletion, E484K, and N501Y variants by BNT162b2 vaccine-elicited sera. Nat Med. (2021) 27:620–1. 10.1038/s41591-021-01270-4 33558724

[101] Dejnirattisai W Zhou D Supasa P Liu C Mentzer AJ Ginn HM. Antibody evasion by the P.1 strain of SARS-CoV-2. Cell. (2021) 184:2939–54e2939. 10.1016/j.cell.2021.03.055 33852911 PMC8008340

[102] Liu Y Liu J Xia H Zhang X Fontes-Garfias CR Swanson KA. Neutralizing activity of BNT162b2-elicited serum. N Engl J Med. (2021) 384:1466–8. 10.1056/NEJMc2102017 33684280 PMC7944950

[103] Parry H Tut G Bruton R Faustini S Stephens C Saunders P. mRNA vaccination in people over 80 years of age induces strong humoral immune responses against SARS-CoV-2 with cross neutralization of P1 Brazilian variant. eLife. (2021) 10:e69375. 10.7554/eLife.69375 34586068 PMC8500710

[104] Puranik A Lenehan PJ Silvert E Niesen MJ Corchado-Garcia J O'Horo JC. Comparison of two highly-effective mRNA vaccines for COVID-19 during periods of Alpha and Delta variant prevalence. medRxiv. (2021). 10.2139/ssrn.390278234401884

[105] Kunal S Aditi Gupta K Ish P. COVID-19 variants in India: Potential role in second wave and impact on vaccination. Heart Lung. (2021) 50:784–787. 10.1016/j.hrtlng.2021.05.008 34217989 PMC8173455

[106] Hossain MK Hassanzadeganroudsari M Apostolopoulos V. The emergence of new strains of SARS-CoV-2. What does it mean for COVID-19 vaccines? Expert Rev Vacc. (2021) 20:635–8. 10.1080/14760584.2021.1915140 33896316 PMC8074646

[107] Chen Y Shen H Huang R Tong X Wu C. Serum neutralising activity against SARS-CoV-2 variants elicited by CoronaVac. Lancet Infect Dis. (2021) 21:1071–2. 10.1016/S1473-3099(21)00287-5 34051887 PMC8159188

[108] Challener CA. BioPharm International. Subunit Vaccines and the Fight Against COVID-19. (2021) Available online at: https://www.biopharminternational.com/view/subunit-vaccines-and-the-fight-against-covid-19 (accessed December 27, 2021).

[109] Karamitros T Papadopoulou G Bousali M Mexias A Tsiodras S Mentis A. SARS-CoV-2 exhibits intra-host genomic plasticity and low-frequency polymorphic quasispecies. J Clin Virol. (2020) 131:104585. 10.1016/j.jcv.2020.104585 32818852 PMC7418792

[110] Jordan SC Shin BH Gadsden TM Chu M Petrosyan A Le CN. (2021). T cell immune responses to SARS-CoV-2 and variants of concern (Alpha and Delta) in infected and vaccinated individuals. Cell Mol Immunol. 18:2554–6. 10.1038/s41423-021-00767-9 34531555 PMC8443898

[111] Naaber P Tserel L Kangro K Sepp E Jürjenson V Adamson A. Dynamics of antibody response to BNT162b2 vaccine after six months: a longitudinal prospective study. Lancet Reg Health Europe. (2021) 10:100208. 10.1016/j.lanepe.2021.100208 34514454 PMC8418937

[112] Levine-Tiefenbrun M Yelin I Alapi H Katz R Herzel E Kuint J. Viral loads of Delta-variant SARS-CoV-2 breakthrough infections after vaccination and booster with BNT162b2. Nat Med. (2021) 2021:1–3. 10.1038/s41591-021-01575-4 34728830

[113] Khoury DS Cromer D Reynaldi A Schlub TE Wheatley AK Juno JA. Neutralizing antibody levels are highly predictive of immune protection from symptomatic SARS-CoV-2 infection. Nat Med. (2021) 27:1205–11. 10.1038/s41591-021-01377-8 34002089

[114] Tarke A Sidney J Methot N Yu ED Zhang Y Dan JM. Impact of SARS-CoV-2 variants on the total CD4(+) and CD8(+) T cell reactivity in infected or vaccinated individuals. Cell Rep Med. (2021) 2:100355. 10.1016/j.xcrm.2021.100355 34230917 PMC8249675

[115] Zuo J Dowell AC Pearce H Verma K Long HM Begum J. Robust SARS-CoV-2-specific T cell immunity is maintained at 6 months following primary infection. Nat Immunol. (2021) 22:620–6. 10.1038/s41590-021-00902-8 33674800 PMC7610739

[116] Ong E Wong M Huffman A He Y. COVID-19 coronavirus vaccine design using reverse vaccinology and machine learning. Front Immunol. (2020) 11:1581. 10.3389/fimmu.2020.01581 32719684 PMC7350702

[117] Jia Q Bielefeldt-Ohmann H Maison RM Maslesa-Galic S Cooper SK Bowen RA. Replicating bacterium-vectored vaccine expressing SARS-CoV-2 membrane and nucleocapsid proteins protects against severe COVID-19-like disease in hamsters. NPJ Vacc. (2021) 6:47. 10.1038/s41541-021-00321-8 33785745 PMC8009914

[118] Batra M Tian R Zhang C Clarence E Sacher CS Miranda JN. Role of IgG against N-protein of SARS-CoV2 in COVID19 clinical outcomes. Sci Rep. (2021) 11:3455. 10.1038/s41598-021-83108-0 33568776 PMC7875990

[119] Jeong H Choi Y-M Seo H Kim B-J. A novel DNA vaccine against SARS-CoV-2 encoding a chimeric protein of its receptor-binding domain (RBD) fused to the amino-terminal region of hepatitis B Virus preS1 with a W4P mutation. Front Immunol. (2021) 12:482. 10.3389/fimmu.2021.637654 33732258 PMC7959807

[120] Noh JY Jeong HW Kim JH Shin E-C. T cell-oriented strategies for controlling the COVID-19 pandemic. Nat Rev Immunol. (2021) 21:687–8. 10.1038/s41577-021-00625-9 34497383 PMC8424399

[121] Shah P Canziani GA Carter EP Chaiken I. The case for S2: the potential benefits of the S2 subunit of the SARS-CoV-2 spike protein as an immunogen in fighting the COVID-19 pandemic. Front Immunol. (2021) 12:508. 10.3389/fimmu.2021.637651 33767706 PMC7985173

[122] de Silva TI Liu G Lindsey BB Dong D Shah D Mentzer AJ. The impact of viral mutations on recognition by SARS-CoV-2 specific T-cells. Cell Rep. (2021) 24:103353. 10.2139/ssrn.3844713 34729465 PMC8552693

