# Supplementary figures and images for: SARS-CoV-2 Mutations and Their Impact on Diagnostics, Therapeutics and Vaccines
Source: Front Med (Lausanne). 2022 Feb 22;9:815389. doi: 10.3389/fmed.2022.815389 (PMC8902153; doi:10.3389/fmed.2022.815389)

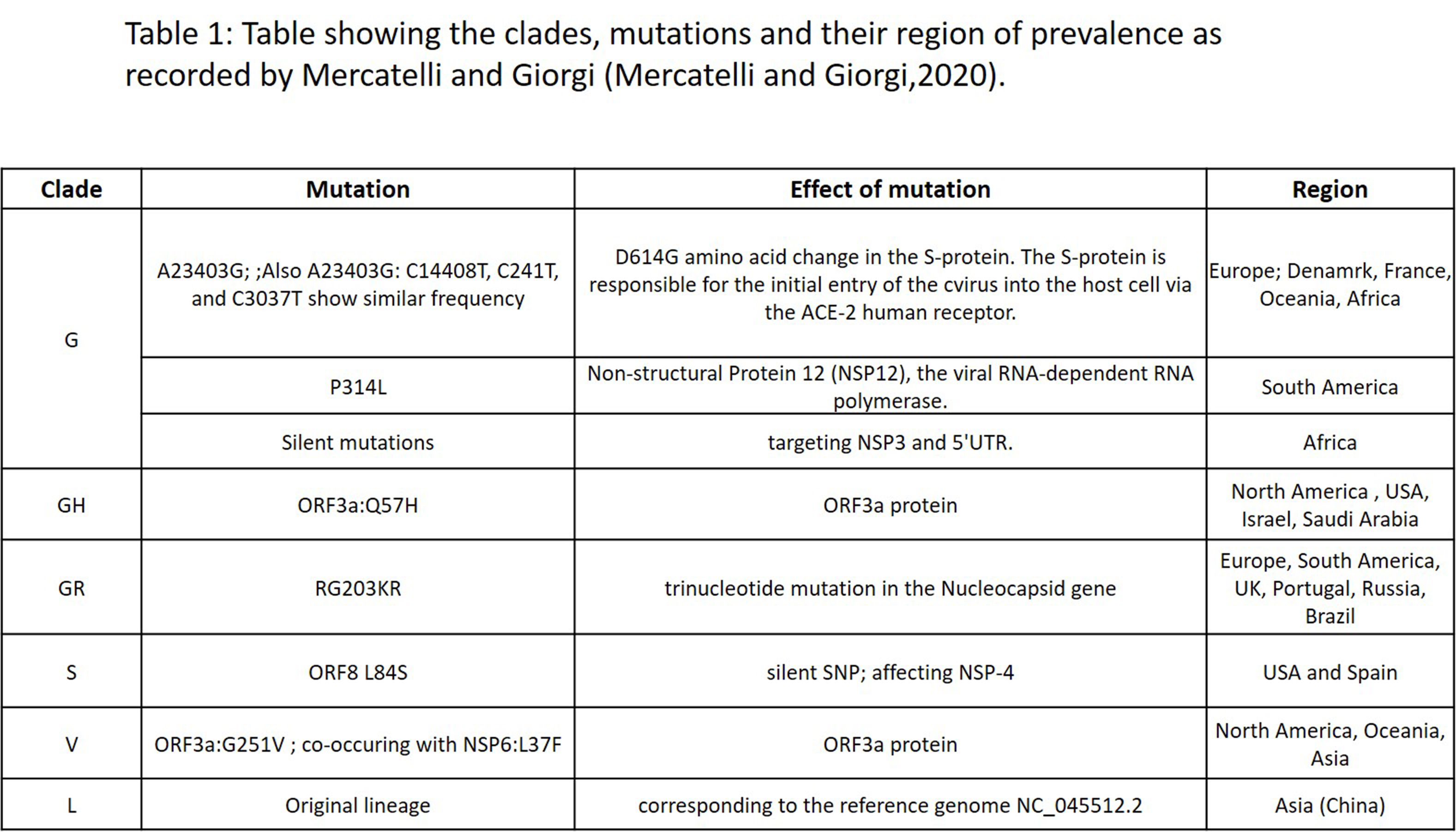

Supplement: Supplementary file 1 [file Image_1.JPEG]

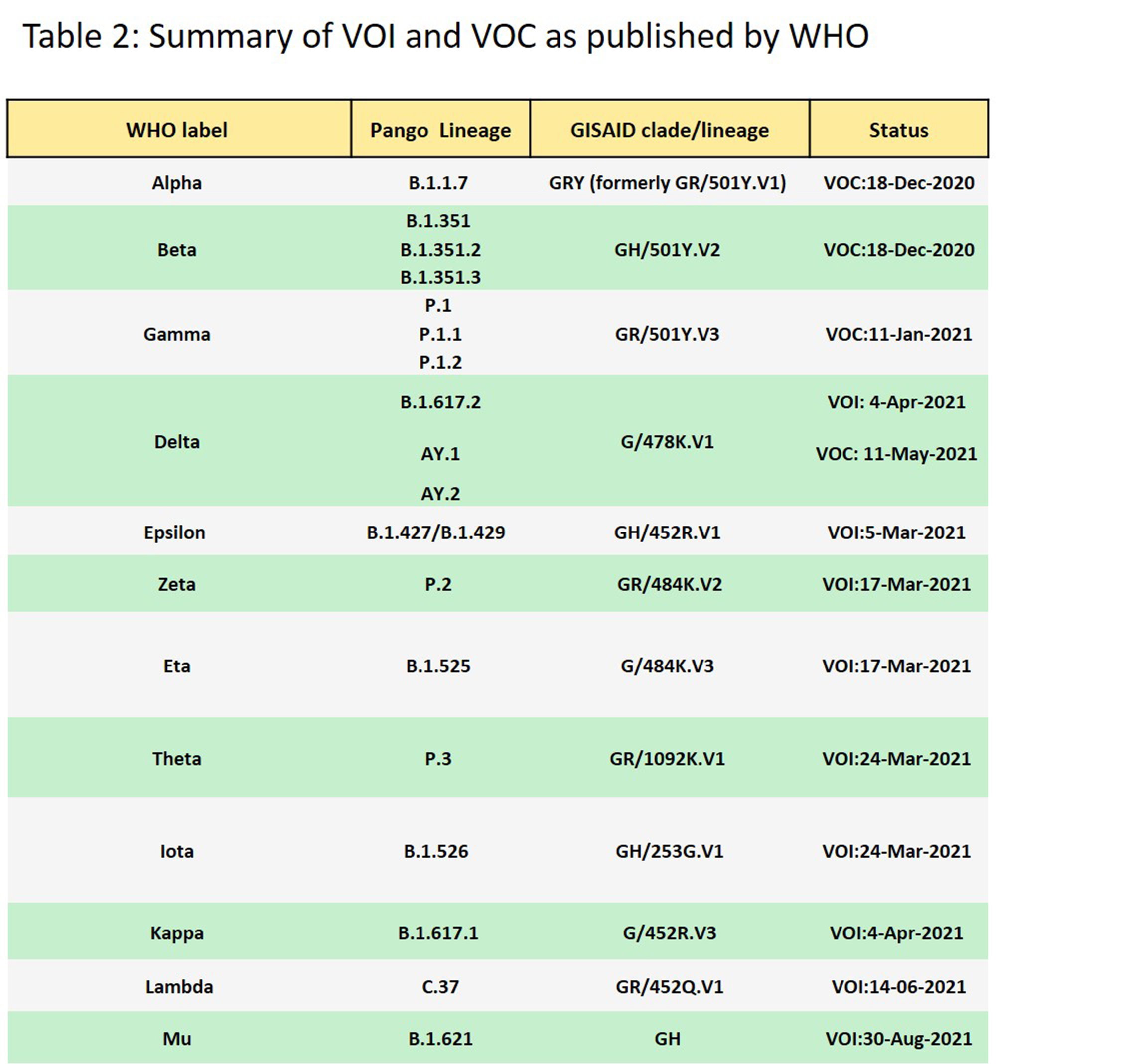

Supplement: Supplementary file 2 [file Image_2.JPEG]

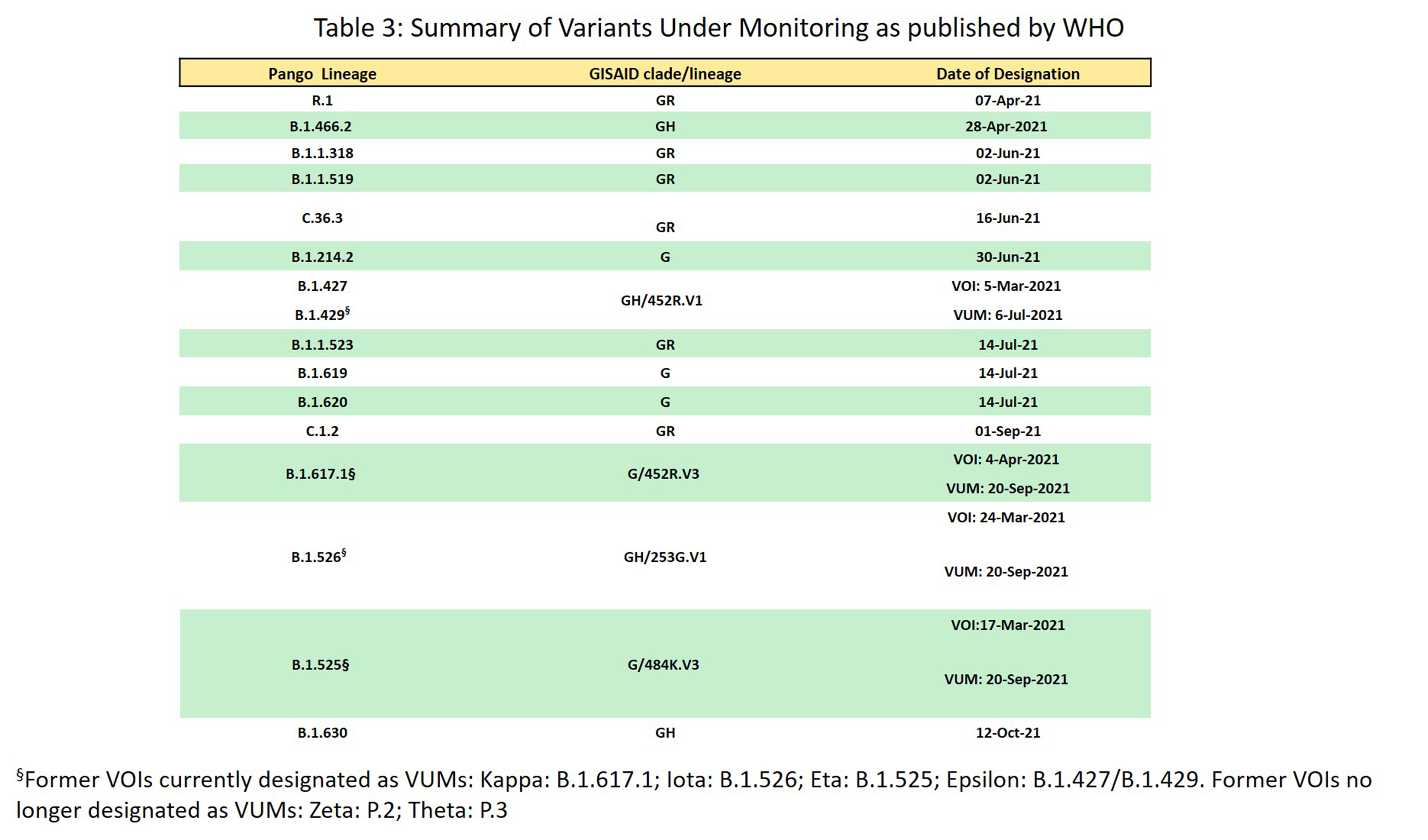

Supplement: Supplementary file 3 [file Image_3.JPEG]

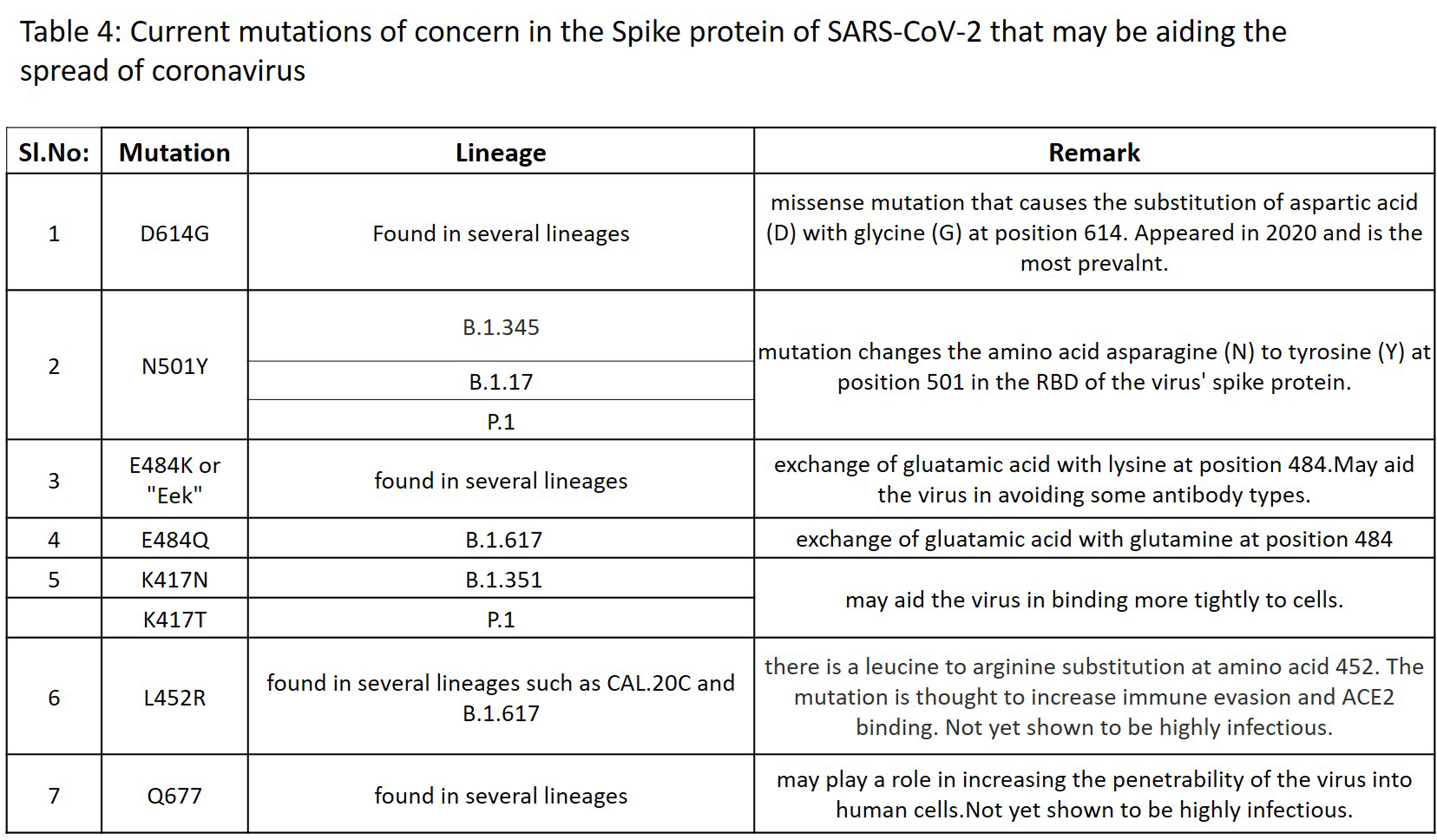

Supplement: Supplementary file 4 [file Image_4.JPEG]

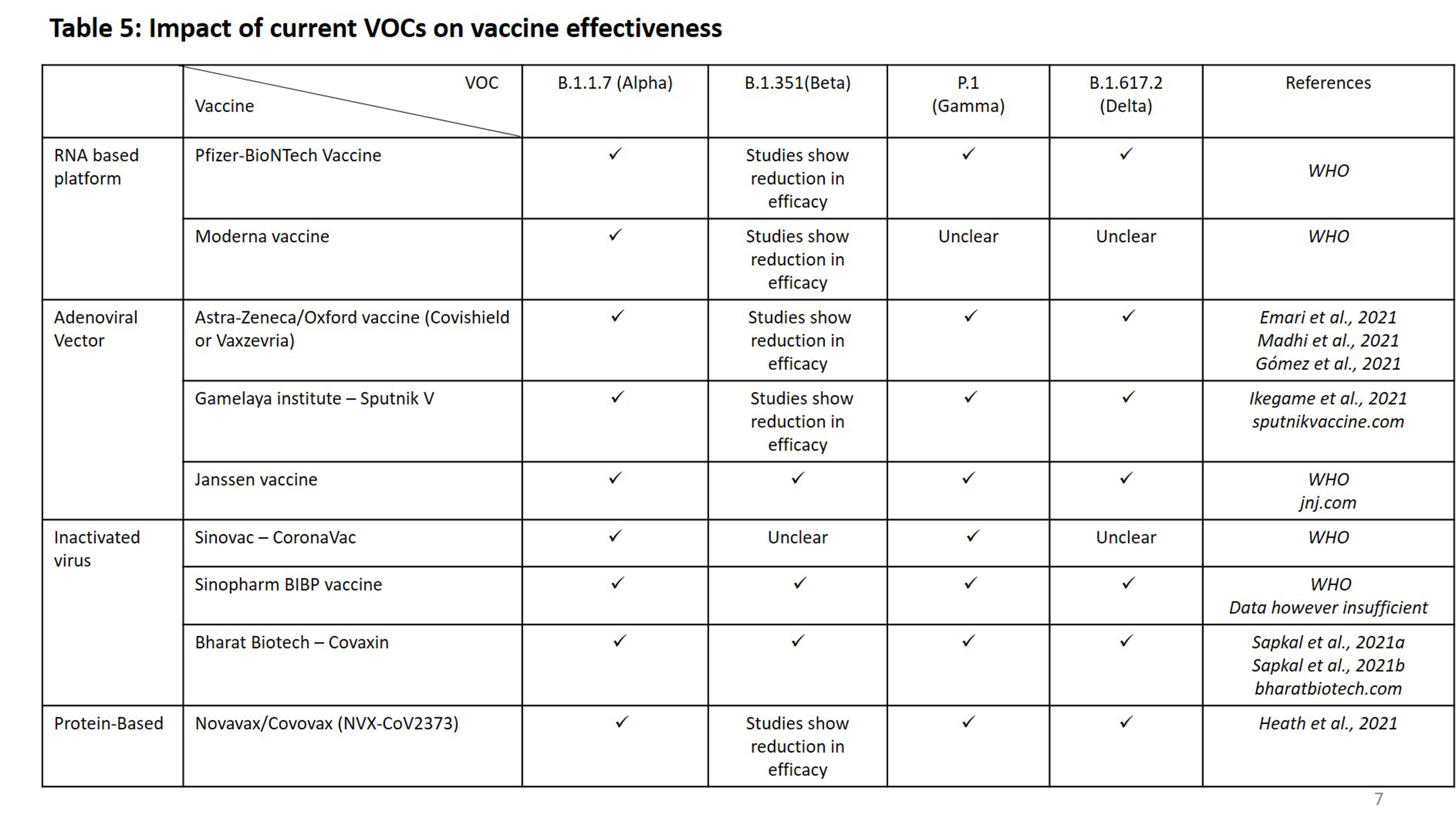

Supplement: Supplementary file 5 [file Image_5.JPEG]
